# Real-time monitoring of the reversible capture and release of CO_2_ on anthraquinone and riboflavin-modified graphitic electrodes using ATR-SEIRAS

**DOI:** 10.1039/d5sc05427c

**Published:** 2025-11-04

**Authors:** Abdur-Rahman Siddiqui, Joel Roberts, Jeanne N'Diaye, Alan L. Ferris, Kristin Martin, Seth T. Putnam, Rohit Bhargava, Jahan Dawlaty, Steven C. Zimmerman, Veronica Augustyn, Joaquín Rodríguez-López

**Affiliations:** a Department of Chemistry, University of Illinois Urbana-Champaign 600 S. Mathews Avenue Urbana Illinois 61801 USA joaquinr@illinois.edu; b The Beckman Institute for Advanced Science and Technology, University of Illinois Urbana-Champaign Urbana Illinois 61801 USA; c Department of Bioengineering, University of Illinois Urbana-Champaign Urbana Illinois 61801 USA; d Department of Materials Science and Engineering, North Carolina State University Raleigh North Carolina 27695 USA; e Department of Chemistry, University of Southern California Chemistry 920 Bloom Walk SSC 702 Los Angeles Los Angeles California 90007 USA; f Department of Chemistry, Ben-Gurion University of the Negev Beer Sheva 8410501 Israel

## Abstract

Electrochemically mediated carbon capture (EMCC) offers a promising alternative to thermochemical carbon capture methods due to its higher energy efficiency and the ability to operate under standard conditions. Graphitic electrodes functionalized with redox-active organic (RAO) capture agents offer a unique opportunity for an inexpensive, abundant, and easily scalable system for direct EMCC. However, the mechanistic understanding and observation of intermediate capture states on carbon surfaces using spectroscopic signatures remains unexplored. Herein, we present *in situ* monitoring of the reversible capture and release of carbon dioxide (CO_2_) from graphene-on-gold electrodes modified with grafted anthraquinone (AQ) and a synthesized riboflavin derivative, 10-ethyl-3-methyl flavin derivative (MFD). These electrodes conveniently enable the use of surface-enhanced infrared absorption spectroscopy in an attenuated total reflectance configuration (ATR-SEIRAS). We successfully decouple the spectral responses of the CO_2_ reduction reaction (CO_2_RR) and CO_2_ capture processes by characterizing AQ- and MFD-modified electrodes under argon and CO_2_ atmospheres. Continuous polarization of the electrode to generate the reduced forms of AQ also generates carbonate (CO_3_^2−^) peaks at 1640 cm^−1^ and 1380 cm^−1^ that overshadow the spectral responses of AQ-bound CO_2_. Conversely, reducing AQ or MFD under inert atmosphere, followed by introducing CO_2_ with no applied potential (*i.e.* at open circuit) results in the growth of distinct spectral features ∼1710–1630 cm^−1^. The release of CO_2_ was observed through the regeneration of an intense carbonyl stretch at ∼1660 cm^−1^ upon electrooxidation, corresponding with CV observations for AQ and MFD. Preliminary CO_2_ capture experiments using a two-electrode flow cell device with AQ- and MFD-modified YP-50 porous carbon electrodes indicate that MFD systems perform on par with AQ systems. This work highlights relevant considerations for performing CO_2_ capture studies under continuous polarization conditions, illuminates key surface intermediates during carbon capture, and demonstrates the ability of our platform to screen novel RAO capture agents.

## Abstract 2

*In situ* ATR-SEIRAS was used to monitor the real-time functionalization and reversible capture and release of CO_2_ at the electrode/electrolyte interface from anthraquinone and 10-ethyl-3-methyl flavin derivative-modified graphitic electrodes.

## Graphical abstract

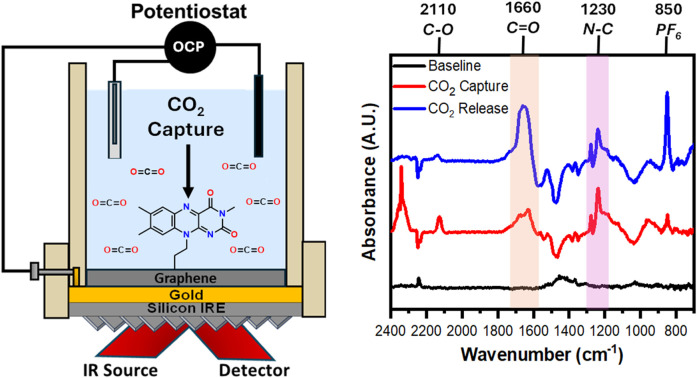

## 1. Introduction

To effectively mitigate the negative impacts of climate change, it is imperative to simultaneously decrease CO_2_ emissions and sequester already-emitted CO_2_ from the atmosphere.^1^ Research efforts for carbon capture have primarily focused on capturing CO_2_ either post-combustion at large industrial point-source emitters^2^ or *via* direct air capture (DAC).^3^ Although DAC is an emerging technology, it is increasingly expected to develop into an essential strategy for attaining net negative carbon emissions in the future.^3,4^ The state-of-the-art methodologies for DAC utilize highly reactive sorbents that selectively absorb CO_2_ such as amines,^5^ alkaline aqueous solutions,^6^ and solid sorbents.^7^ However, all these systems suffer from large energetic costs due to their inefficient regeneration, *i.e.*, CO_2_ desorption, through temperature and/or pressure swings.^5–8^ Thus, electrochemically mediated carbon capture (EMCC) has emerged as a new front-runner due to its scalability, higher energy efficiencies, and the ability to operate under ambient conditions.^8–10^ EMCC methodologies vary between direct binding of CO_2_ to a redox-active organic (RAO) molecule in aprotic solvents, or indirect capture *via* shifting the pH of aqueous systems using a proton-coupled electron transfer (PCET) reaction; this study will focus on the former.^8–12^

In direct EMCC, a RAO molecule reversibly captures and releases CO_2_ by electrochemical modulation of the RAO molecule's affinity for CO_2_ complexation.^8–12^ In general, an RAO capture agent (**R**) is reduced in an aprotic solvent to form a highly reactive nucleophilic species (**R**^***n*−**^) (Reaction (1)). This **R**^***n*−**^ species has an increased CO_2_ binding affinity such that a spontaneous nucleophilic attack forms a stable CO_2_ adduct (**R–CO**_**2**_^***n*−**^) (Reaction (2)). To release the bound CO_2_, the **R–CO**_**2**_^***n*−**^ complex is oxidized into a neutral species (**R–CO**_**2**_) (Reaction (3)), which has a drastically lower affinity for CO_2_ complexation, resulting in subsequent desorption of CO_2_ and the regeneration of **R** species (Reaction (4)).^8–12^ It is important to note that as the difference in CO_2_ binding affinity grows between **R** and **R**^***n*−**^, the potential difference between capture and release grows as well, increasing the overall energetic requirement of the system.^13^ The ideal redox-active capture agent should have sufficient CO_2_ binding affinities and fast electron kinetics while minimizing the potential difference between capture and release events.^8–12^ In literature, the most broadly investigated RAO capture agents are quinones,^14–17^ but the search continues for other capture agents such as thiols,^18^ amines,^19,20^ metal–organic frameworks,^21^ and heterocycles.^22,23^

1 **R** + *n*e^−^ ⇋ **R**^***n***−^

2 **R**^***n***−^ + CO_2_ → **R**–CO^***n***−^_2_

3 **R**–**CO**^***n***−^_2_ ⇋ **R**–**CO**_2_ + *n*e^−^

4 **R**–CO_2_ → **R** + CO_2_

There is a growing focus on developing viable carbon capture devices by immobilizing RAO capture agents onto electrode materials for increased CO_2_ capture capacity and device lifetimes.^24–28^ Graphitic electrodes functionalized with RAO capture agents offer a unique opportunity for an inexpensive, abundant, electronically conductive, and easily scalable system for EMCC.^25^ However, *in situ* tracking of the capture and release of CO_2_ from a graphitic electrode functionalized with RAOs remains largely unexplored. Combining electrochemistry and spectroscopy allows for the discernment of interfacial processes at electrode surfaces. Electrochemical attenuated total reflectance surface-enhanced infrared absorption spectroscopy (ATR-SEIRAS) has been previously used for validating the carbon capture mechanisms of quinones in solution^28–30^ and screening polymers of RAO capture agents deposited on Au electrodes,^31^ but not for probing highly desirable graphitic interfaces functionalized with RAO capture agents.

Herein, we exploit the expanded capabilities of our previously developed graphene-on-gold electrodes^32^ to perform electrochemical ATR-SEIRAS on a graphitic interface. These graphene-on-gold electrodes enable tracking the functionalization of the graphitic surface with higher spectral clarity than those of traditional plasmonic metal electrodes.^32^ We posited that these electrodes would allow us to monitor the functionalization and reversible capture and release of CO_2_ at the electrode/electrolyte interface in real-time. Moreover, methods for functionalizing graphitic electrodes *via* diazonium chemistries are well-documented^33–35^ especially for the known RAO capture agent, anthraquinone (AQ).^25,36–46^ Thus, AQ-modified graphene-on-gold electrodes were used as a benchmark system for demonstrating the ability of the graphene-on-gold platform to observe interfacial carbon capture.

Additionally, we utilized our platform to investigate vitamin B_2_ as a novel RAO capture agent. Vitamin B_2_ or riboflavin (RF) is a heterocyclic precursor to two biological coenzymes (FAD and FMN). The heterocyclic unit itself is capable of multiple PCET reactions in metabolic pathways.^47–50^ RF has also been used for indirect EMCC in a pH-swing flow device.^51^ The two-step electrochemical reduction of RF in aprotic solvents was shown to produce a reactive nucleophilic species (RF^2^),^52–54^ however, this is the first study to demonstrate that RF^2−^ binds CO_2_. Unlike AQ, current methods for functionalizing carbon electrodes with RF are limited and may be difficult to implement in CO_2_ capture.^56,57^ Thus with RF as the starting material, herein we synthesize and use a flavin derivative, 10-ethyl-3-methyl flavin derivative (MFD), to facilitate the generation and electroreduction of diazonium functional groups for straightforward electrode modification. In this work, we show the binding and release of CO_2_ on AQ-functionalized and MFD-functionalized graphene-on-gold electrodes, focusing on CO_2_ adduct formation and their adsorption/desorption kinetics. Lastly, the CO_2_ capture performances of functionalized YP50 electrodes were determined with a custom flow cell setup for cycling the electrodes under a saturated atmosphere while tracking the changes in CO_2_ concentration.

## 2. Materials and methods

### 2.1. Materials

All starting materials and solvents were obtained from commercial sources and used without further purification. For synthesis, we procured riboflavin (98%, Ambeed), periodic acid (99+ %, Thermo Scientific), hydrochloric acid (36.5–38.0%, Fisher Scientific), sodium bicarbonate (99.7%, VWR), acetic acid (99.5–100.5%, J.T. Baker), benzylamine (99.0%, Tokyo Chemical Company), platinum black (H-TEC, education), sulfuric acid (95–98%, Fisher Scientific), ethyl acetate (99.9%, Fisher Scientific), methanol (99.8%, Fisher Scientific), diethyl ether (99%, Fisher Scientific), ethanol (200 proof, Decon Laboratories, Inc), di-*tert*-butyl dicarbonate (99.5%, Chem-Impex INT’L INC), triethylamine (99%, Thermo Scientific), magnesium sulfate (97.8–100.3%, Fisher Scientific), chloroform (99.8%, Fisher Scientific), potassium carbonate (99.0%, VWR), dimethylformamide (99.8%, Thermo Scientific), methyl iodide (99.5%, Thermo Scientific), methylene chloride (99.9%, Fisher Scientific), ethylenediaminetetraacetic acid disodium dehydrate salt (EDTA, Millipore Sigma), 1-octanol (99%, Millipore Sigma), hexanes, mixture of isomers (98.5%, Millipore Sigma), 2-aminoanthraquinone (2-AAQ, 85%, Millipore Sigma), acetonitrile (MeCN, 99%, Millipore Sigma), *t*-butyl nitrite (90%, Millipore Sigma), tetrabutylammonium hexafluorophosphate (TBAPF_6_, 98%, Millipore Sigma), gold(iii) chloride trihydrate (99.9%, Millipore Sigma), poly(bisphenol A carbonate) (Millipore Sigma), acetone (99.5%, Fisher Scientific), 2-propanol (99.8%, Honeywell), and copper etchant type CE–100 (Transene). Aqueous solutions were made using deionized water from a Millipore Sigma Direct-Q 8 (resistivity of 18.2 MΩ). UHP-grade argon and industrial-grade CO_2_ (Airgas) were used in all ATR-SEIRAS experiments. For the flow cell electrodes, we used YP-50 activated carbon (Kuraray), titanium gauze (100 mesh woven from 0.05 mm diameter wire, Fisher Scientific), acetylene black (100% compressed, Fisher Scientific), polytetrafluoroethylene (PTFE; 60 wt% dispersion in water, Millipore Sigma), ethanol (200 proof, Fisher Scientific). The flow cell utilized titanium foil current collectors (0.127 mm thick, Fisher Scientific). The flow cell utilized 20% CO_2_/balance argon gas (ARC3). The flow cell electrolyte consisted of lithium bis(trifluoromethanesulfonyl)imide (LiTFSI, 98.0%, Fisher Scientific) and propylene carbonate (PC, 99.0%, anhydrous, Millipore Sigma). The flow cell utilized a polypropylene separator (Celgard 3501).

### 2.2. IR instrumentation

ATR-SEIRAS experiments were performed utilizing a PerkinElmer Spectrum 3 FTIR with a liquid nitrogen-cooled MCT detector. The FTIR was fitted with a VeeMax III (PIKE technologies) specular reflection accessory and coupled with a Jackfish electrochemical cell (PIKE technologies). The specialized internal reflection element (IRUBIS) was used with a 35° angle of incidence.^55^ The spectra were collected between 4000–600 cm^−1^ at 4 cm^−1^ resolution using an 8.94 mm J-stop with a scan speed of 1 cm^−1^ s^−1^. For SEIRAS measurements the scan accumulation was 56 scans except for time-resolve experiments where it was 9.

### 2.3. ATR-SEIRAS setup and substrate

All electrochemical ATR-SEIRAS experiments used a CHI 1242C potentiostat (CH Instruments Inc.) with a carbon rod for the counter electrode and an Ag/AgCl reference electrode (3 M KCl) connected *via* a 0.1 M NaClO_4_|agar salt bridge. The internal reflection element and working electrode used for all ATR-SEIRAS experiments was a graphene-on-gold substrate developed in an earlier study.^32^

### 2.4. Synthesis of the 10-(2-ammoniumethyl)-3-methyl flavin derivative (AMFD)

The synthetic route (Scheme S1) and procedures used to derive the AMFD are detailed in the SI (Section 1).

### 2.5. YP50 electrode preparation, functionalization, and flow cell setup for tracking CO_2_ adsorption

The procedures for preparing YP-50 carbon electrodes, functionalizing with AQ and MFD, and the flow cell setup (Schemes S2 and S3) are detailed in SI (Section 2).

## 3. Results and discussion

### 3.1. Monitoring the electrografting of anthraquinone on graphene-on-gold electrodes

**Scheme 1:**
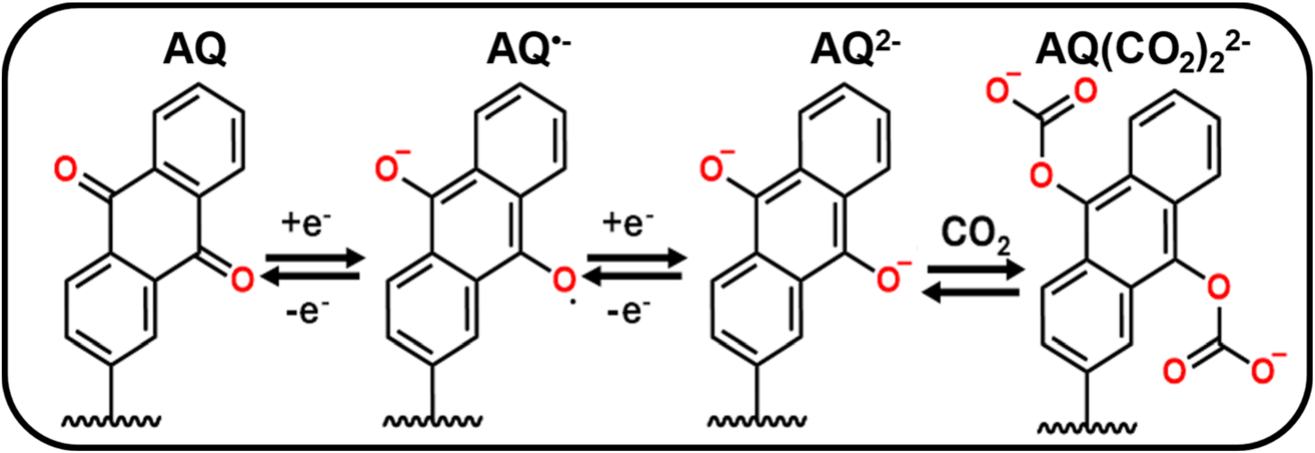
Mechanistic depiction of the electrochemical reduction of AQ and subsequent binding to CO_2_ through an EECC reaction mechanism.

Scheme 1 depicts the two-step electrochemical reduction of AQ in deoxygenated non-aqueous solvents, first forming a radical anion species (AQ˙^−^), and subsequently the dianion (AQ^2−^).^13,25^ When AQ^2−^ is exposed to CO_2_, it spontaneously reacts and binds with this species to form two CO_2_-adducts (AQ-(CO_2_)_2_^2−^).^13,25^ This binding was predominantly reported to occur *via* an EECC mechanism (E = electrochemical, C = chemical), although an ECEC mechanism was also reported, with AQ˙^−^ binding to CO_2_ before the second reduction (Scheme S4).^8^ Regardless of the mechanism, the primary product would be the final product shown in Scheme 1.

**Fig. 1:**
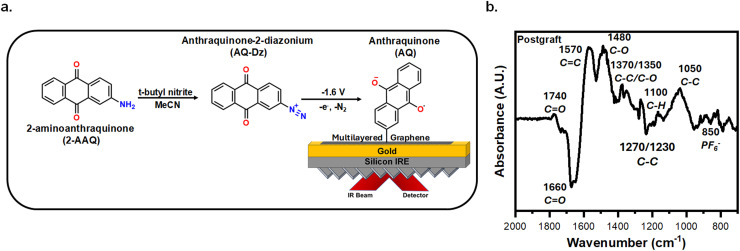
Electrografting AQ onto graphene-on-gold electrodes. (a) The synthetic route for converting 2-aminoanthraquinone (2-AAQ) to anthraquinone-2-diazonium (AQ-Dz) followed by electrografting of AQ onto a graphene-on-gold substrate. (b) A labelled spectrum detailing the peaks observed after the graft. The reference spectrum was taken at 0.4 V in 0.1 M TBAPF_6_ in MeCN with 1 mM AQ-Dz.

Fig. 1a depicts a schematic for generating anthraquinone-diazonium (AQ-Dz) and the subsequent electrografting onto a graphene-on-gold substrate. The AQ-Dz was generated by mixing 2-aminoanthraquinone (2-AAQ) with *t*-butyl nitrite in 0.1 M TBAPF_6_ in MeCN for at least 20 minutes.^25,39–44^ Next, the generated AQ-Dz was electrografted onto a graphene-on-gold electrode under an argon purge by holding a potential of −1.6 V (*vs.* Ag/AgCl) for 10 minutes to create reducing conditions for generating the reactive intermediate from AQ-Dz. The reduction of the diazonium followed by N_2_ loss forms an aryl radical that terminates on the graphitic face of the electrode, thus covalently attaching the AQ moiety to the graphite surface.^34^ Moreover, at this potential the AQ undergo a one-electron reduction into AQ˙^−^ (Scheme 1). Fig. S1a tracks the *in situ* functionalization of the electrode in real-time using ATR-SEIRAS. The reference spectra used for electrografting was acquired by holding 0.4 V in 0.1 M TBAPF_6_ in MeCN with 1 mM AQ-Dz, a potential where AQ is the dominant species. The peak assignments, shown for the postgraft electrode in Fig. 1b, were determined from previously reported spectroscopic AQ studies (Table S1).^27,28,31,46,56–60^ The main peaks observed during the graft are the positively growing 1570 cm^−1^ C=C band, 1480 cm^−1^ C–O stretch, 1370/1350 cm^−1^ C–C/C–O stretch, 1270/1230 cm^−1^ C–C stretch, the 1100 cm^−1^ C–H stretch, the 1050 cm^−1^ C–C band, and the 850 cm^−1^ band caused by the PF_6_^−^ counter ion in the electrolyte. A small peak observed at 1740 cm^−1^ emerges solely during the electrografting, which we hypothesize corresponds to the unreacted AQ species in the grafting solution. A large negatively growing peak was observed at 1660 cm^−1^ which correlates to a decrease in the intensity of the C=O stretch from the quinone AQ species being reduced to the semiquinone AQ˙^−^ species which leads to the growth of the 1480 C–O band.^46^ After grafting, a progression of the intensity of this band as a function of potential is shown in Fig. S1b, confirming its association to the formation of AQ˙^−^.

### 3.2. Probing the redox-behavior of AQ-modified graphene-on-gold under argon and CO_2_

**Fig. 2:**
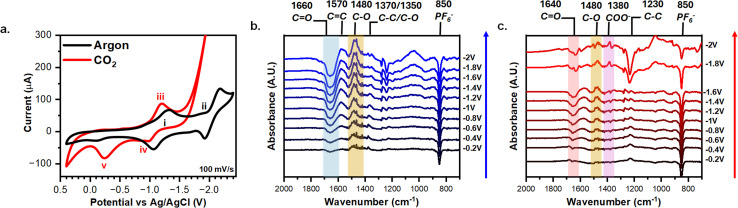
CVs and ATR-SEIRAS characterization of AQ-modified graphene-on-gold electrodes in 0.1 M TBAPF_6_ MeCN. (a) CVs of AQ-modified graphene-on-gold under argon and CO_2_ purge with key peaks labelled i–v. Potential-dependent spectra AQ-modified graphene-on-gold under (b) argon or (c) CO_2_ purge with key peak highlighted and labeled. The reference spectra were taken at 0.4 V under either argon or CO_2_ saturation.

The AQ-modified graphene-on-gold was rinsed thoroughly, and cyclic voltammograms (CV) were taken in MeCN with 0.1 M TBAPF_6_ to verify the graft electrochemically. Fig. 2 illustrates the redox behavior of AQ-modified graphene-on-gold under argon and CO_2_ purges. Under argon, the CV displays two distinct redox peaks at −1.28 V (i) and −2.01 V (ii) with peak-to-peak separation of 0.15 V and 0.20 V, respectively (Fig. 2a). Despite the large peak-to-peak separation, the attachment of the AQ can be verified by exploring the dependency of peak current *versus* scan rate. Fig. S2a shows the CV of the AQ-modified graphene-on-gold electrode under argon at different scan rates (15–200 mV s^−1^). Both the anodic and cathodic peak currents linearly correlate slightly better with scan rate (surface confined process) compared to the square root of scan rate (diffusive process), suggesting the redox observed is from a surface-bound species (Fig. S2b). The quality of this assessment could be complicated by the formation of a multilayer during the grafting process. The average surface coverage obtained was 1.5 ± 0.3 nmol cm^−2^ which is in good agreement with the lower bound for the formation of a multilayer film of AQ on a graphite surface reported by Bousquet *et al.* (1.4–10.3 nmol cm^−2^).^37^

The CV of the attached AQ drastically alters when exposed to CO_2_. A first reduction peak appears slightly shifted with respect to that in the absence of CO_2_ (iii) (−1.20 V *vs.* −1.28 V, respectively). However, the second reduction peak (ii) seems to disappear, giving way to an exponentially increasing current beyond −1.8 V which we ascribe to the CO_2_ reduction reaction (CO_2_RR) as long as potential is continuously applied (Fig. 2a).^61,62^ Acetonitrile has been previously demonstrated to govern the products formed from CO_2_RR to be carbonate (CO_3_^2−^) and dissolved carbon monoxide (CO), regardless of the electrode material used *via* a disproportionate reaction between two CO_2_ molecules (Reaction (5)).^63^

5 2CO_2_ + 2e^−^ → CO^2−^_3_ + CO

We also note that there is an increase in the current intensity of the CV peak in the presence of CO_2_ (iii) in Fig. 2a (and its area under the curve), compared to the measurement in argon. This suggests the merging of the CV response of the peaks i and ii into a single peak, which has been proposed to result from making the second reduction more thermodynamically favorable when CO_2_ is bound.^13,25^ On the reverse scan of the CV, we observed a small oxidation peak (iv) at −1 V correlating to the oxidation of AQ^2−^ which presumably did not bind CO_2_, possibly due to the formation of a multilayer of AQ. On the other hand, an oxidation peak (*v*) that is consistent in intensity with the forward-going peak was observed at −0.25 V indicating the oxidization of AQ(CO_2_)_2_^2−^ and the release of CO_2_ (Fig. 2a) at a more positive potential than for the oxidation of AQ^2−^ and AQ˙^−^.^13,25^

We now turn to exploring the changes at the electrochemical interface under argon and CO_2_ using ATR-SEIRAS. Spectra were referenced at 0.4 V to better resolve the changes due to the redox behavior of the AQ. Each spectrum was acquired using chronoamperometry for 120 s Fig. 2b shows how the 1660 cm^−1^ C=O (blue shaded) and 1270/1230 cm^−1^ C–C bands grow negatively at progressively negative potentials below −0.8 V while the 1570 cm^−1^ C=C, 1480 cm^−1^ C–O (yellow shaded), 1370/1350 cm^−1^ C–C, and the 1050 cm^−1^ C–C bands grow positively. Our results coincide with a recent study by Burgess *et al.* in which the peaks at 1666 cm^−1^, 1480 cm^−1^, and 1360 cm^−1^ are attributed to AQ, AQ˙^−^, AQ^2^, respectively.^46^ As observed during the electrografting, the negatively growing 1660 cm^−1^ C=O band and the positively growing 1480 cm^−1^ C–O peak are a direct observation of the reduction of AQ into the semiquinone species AQ˙^−^. Moreover, at −1.8 V the reduction of AQ˙^−^ to AQ^2−^ is observed *via* the splitting of the 1360 cm^−1^ ring mode band into two bands at 1370 cm^−1^ and 1350 cm^−1^ stretches. Fig. 2c shows the changes in the spectra under CO_2_ compared to argon. A new reference spectrum was taken at 0.4 V under CO_2_ purge to better distinguish the changes in the IR bands corresponding to the binding of CO_2_. In both argon and CO_2_, the 850 cm^−1^ from PF_6_^−^ turned negative, showing the expected depletion of the electrolyte anion at a negatively charged electrode. Under CO_2_, we observed that at −1.8 V and −2 V the 1230 cm^−1^ C–C peak goes negative and the 1640 cm^−1^ C=O (red shaded) and 1380 cm^−1^ COO (purple shaded) peaks emerge. However, these 1640 cm^−1^ and 1380 cm^−1^ peaks were observed for CO_3_^2−^ generated *via* CO_2_RR.^63^ Since CO_2_RR is an inner-sphere process, CO_2_ must be adsorbed to the electrode surface for the reaction to occur, thus our SEIRAS platform enhances the signal from these formed products.^63,64^ However, this signal overshadows any signal stemming from CO_2_ capture and complicates spectral analysis. Thus, a different experimental approach is required to effectively differentiate between signals derived from CO_2_ capture and those from CO_2_RR products.

### 3.3. Decoupling CO_2_RR and CO_2_ capture using an AQ-modified electrode

**Fig. 3:**
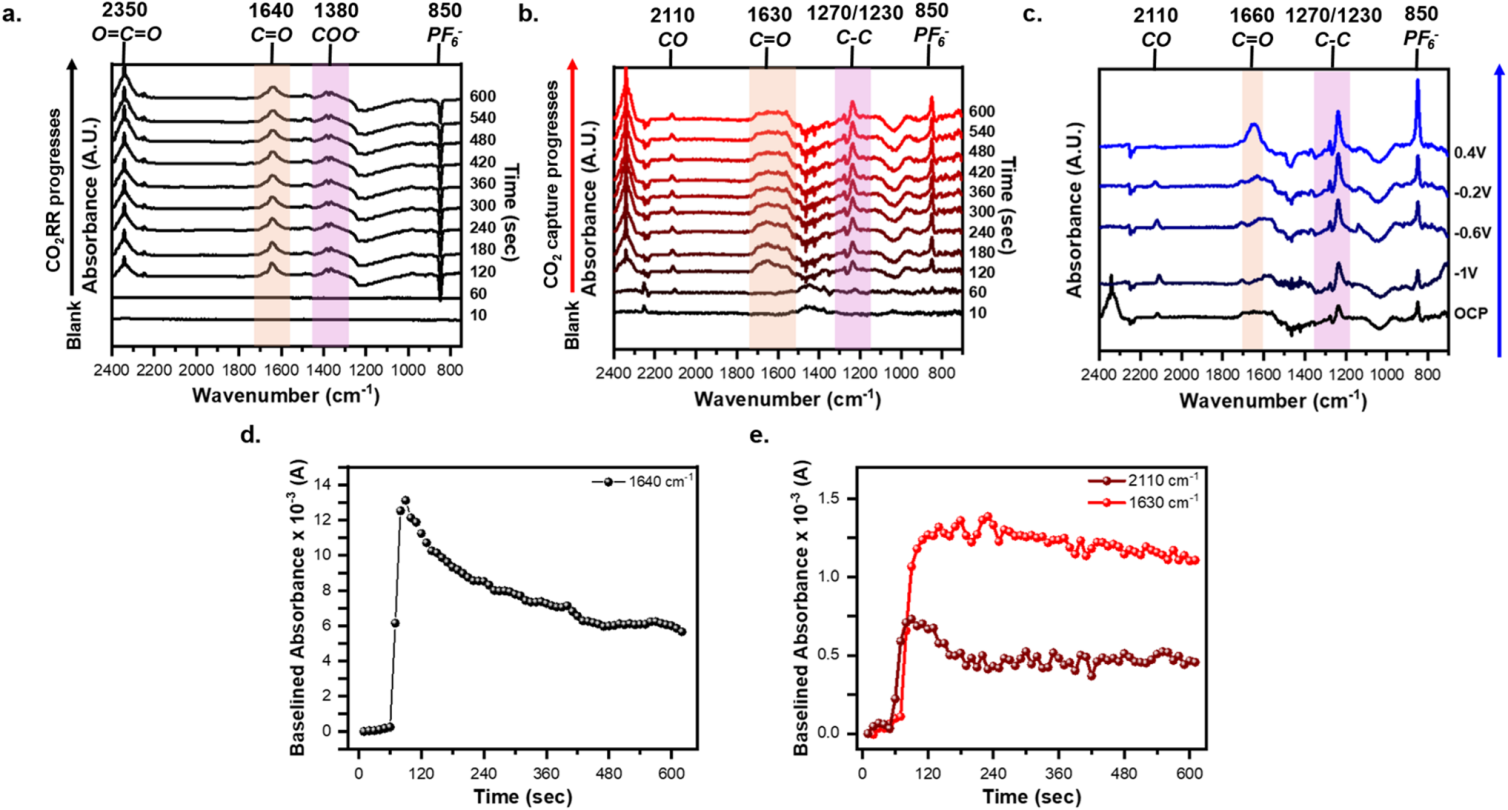
Time-resolved spectra of the CO_2_RR, CO_2_ capture, and release of CO_2_ using AQ-modified graphene-on-gold with key peak highlighted and labeled. (a) *In situ* monitoring of CO_2_RR by holding −2.1 V for 10 min. (b) *In situ* monitoring of the CO_2_ capture by holding −2.2 V for 1 min under argon purge, then 9 min of ambient CO_2_ capture without any applied potential. (c) The release of the CO_2_ is observed when holding progressively positive potentials for 10 min each under argon purge. Tracking the baselined absorbance value of the (d) 1640 cm^−1^ peak during CO_2_RR (e) the 2110 cm^−1^ and 1630 cm^−1^ peak during CO_2_ capture. The reference spectra was taken at −2.1 V and −2.2 V under argon purge for CO_2_RR and CO_2_ capture, respectively. The spectra at 10 s were used as the baseline.

*In situ* monitoring of the CO_2_RR and CO_2_ capture was performed by taking time-resolved spectra with the AQ-modified electrode. To this point, we first established the role of CO_3_^2−^ in the observed signal. The reference spectra were the AQ-modified electrode held at −2.1 V under argon to form AQ^2−^ to ensure any spectral changes are due to AQ^2−^ binding to CO_2_ or formation of CO_3_^2−^. Fig. 3a shows the spectral response when intentionally causing CO_2_RR to occur by holding −2.1 V throughout the entire experiment. For the first 60 s the cell remained under an argon purge, thus displaying no difference to the reference spectra. Yet, upon switching to CO_2_ after 60 s CO_2_RR occurs immediately producing the 1640 cm^−1^ (red shaded) and the 1380 cm^−1^ (purple shaded) CO_3_^2−^ peaks. These peaks are assigned to CO_2_RR as replicating the same experiment with an unmodified graphene-on-gold electrode elicits a similar spectral response (Fig. S3). Fig. 3d shows that the baselined 1640 cm^−1^ peak height spiked as soon as CO_2_ was purged into the cell, then steadily declined over time. This suggests a kinetic limitation arising from the mass transfer of the continuously produced CO_3_^2−^.^63,65^ Further evidence for these peaks arising from continuous CO_2_RR is that holding the potential positive of −1.8 V does not generate the peaks at 1640 cm^−1^ and 1380 cm^−1^ (Fig. S4) and when the applied potential at −2.1 V is suddenly interrupted, these peaks decrease immediately (Fig. S5). From these experiments we establish that CO_2_RR produces CO_3_^2−^ on the surface of the electrode which dominates the spectral response, and thus a different strategy is needed.

To avoid complications from CO_2_RR during the capture event, we turned to reducing the immobilized-AQ followed by interruption of the applied potential and subsequent introduction of CO_2_ to enable the capture to take place at the open circuit potential (OCP). This ambient capture of CO_2_ is enabled by the EECC reaction mechanism in which AQ^2−^ chemically reacts with CO_2_ (Scheme 1), thus no applied potential is required beyond the initial reduction of the AQ to AQ^2−^. To accomplish this the AQ was first reduced to AQ^2−^ under argon purge by holding −2.2 V for 1 min, then applied bias was removed and CO_2_ was purged for 9 min. The spectral response differs drastically to that of the CO_2_RR (Fig. 3b*vs.*Fig. 3a). The key differences in the spectroscopic responses are the growth of a very broad peak from 1710 to 1560 cm^−1^ (red shaded) centered around 1630 cm^−1^ which we ascribe to the formation of AQ(CO_2_)_2_^2−^ species on the electrode surface. This broad peak may be rationalized by the overlap of various signals, since the range from 1710 to 1600 cm^−1^ corresponds to C=O stretches of bound-CO_2_ while the 1600–1560 cm^−1^ corresponds to C=C stretches of the AQ ring. Other peaks observed are positively growing at 2350 cm^−1^*ν*_as_(O=C=O) from CO_2_ dissolved in solution, 2110 cm^−1^ from CO, 1270/1230 cm^−1^ assigned to C–C (purple shaded), and 850 cm^−1^ PF_6_^−^. In contrast with continued polarization to drive the CO_2_RR, on an unmodified graphene-on-gold electrode, no such broad peak from 1710–1560 cm^−1^ was observed (Fig. S6).

The peak at 2110 cm^−1^ was correlated to adsorbed CO on the electrode surface as a product of CO_2_RR.^63^ Yin *et al.* previously reported that AQ-functionalized electrodes can indirectly mediate CO_2_RR forming CO_3_^2−^ and CO.^61^ The formation of CO_3_^2−^ and CO could help explain the broad peak which contains contribution from both the bound-CO_2_ (1710–1660 cm^−1^) and any formed CO_3_^2−^ (1640 cm^−1^). However, a separate *in situ* ATR-SEIRAS investigation of polyanthraquinone coupled with density functional theory (DFT) simulations attributed peaks between 2200–2100 cm^−1^ to CO_2_ adsorbed onto RAO polymers.^31^ Tracking the peak heights at 2110 cm^−1^ and 1630 cm^−1^ shows an instantaneous spike in the peak height upon introduction of CO_2_ to the electrochemical cell followed by a plateauing within the first 3 minutes (Fig. 3e). This demonstrates the reaction between CO_2_ and AQ^2−^ in real-time. In particular, the 1630 cm^−1^ peak which we ascribe to the captured CO_2_ showed minimal decrease with time after reaching its plateau, suggesting it is a stable species on the electrode even without applied potential.

To release the bound CO_2_, the AQ-modified electrode was subsequently held at −1 V, −0.6 V, −0.2 V, and 0.4 V for 10 min each under argon purge (Fig. S7). We chose these potentials as they align with distinct features in the CV shown in Fig. 2a, with potentials more negative than −0.6 representing areas where the bound form is likely to be stable and those more positive where oxidation of the bound form and release of CO_2_ should be observed. Fig. 3c demonstrates how the broad postcapture peak between 1710–1560 cm^−1^ begins to change with increasingly positive applied potential. At −1 V, the broad feature splits into three distinct peaks at 1705 cm^−1^, 1630 cm^−1^, and 1590 cm^−1^ while the 1270/1230 cm^−1^ C–C peak grows in intensity. We believe that these changes arise because of the different electrostatic environment at this potential, possibly re-orienting the molecules at the surface.^66^ At −0.6 V and −0.2 V, the 2110 cm^−1^, 1630 cm^−1^, 850 cm^−1^ peaks continually grow, but qualitative changes are small. Finally at 0.4 V, beyond the CV oxidation feature observed in the presence of CO_2_ in Fig. 2a, the peak immediately transforms into a characteristic 1660 cm^−1^ feature corresponding to the quinone form of AQ, implying the full oxidation of AQ(CO_2_)_2_^2−^ back to AQ and the release of CO_2_.^46^ Moreover, the 850 cm^−1^ PF_6_^−^ peak shows a sudden increase, which suggests the removal of excess charge brought by the surface-bound AQ(CO_2_)_2_^2−^. Finally, it is noteworthy that the 2110 cm^−1^ peak, ascribed to the formation of carbon monoxide or bound CO_2_ (see above) also disappears during this last step to 0.4 V. We speculate that retention of this species could be also related to the redox state of AQ. We are unaware of experiments attempting the capture of CO using AQ, but this is a possibility that could be explored in the future. Overall, our time, potential and condition resolved experiments support the capture and release of CO_2_ at a graphitic interface.

### 3.4. Assessing riboflavin for direct electrochemically mediated carbon capture

**Scheme 2:**
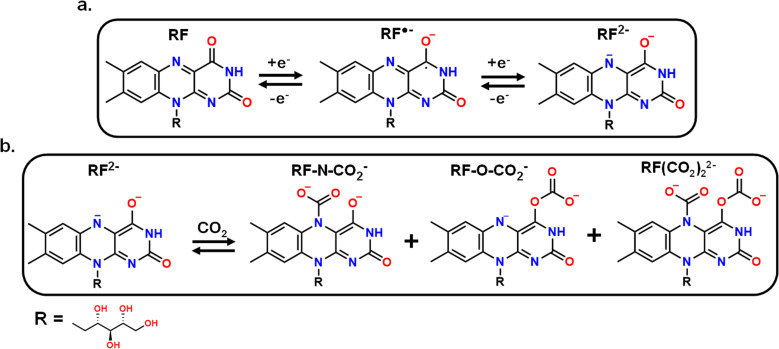
(a) Previously reported mechanism for the two-step electroreduction of riboflavin in aprotic solvents. (b) Proposed reaction scheme for reduced RF (RF^2−^) with CO_2_ to form three separate products.

With a new methodology in hand to determine the potential-dependent capture of CO_2_ at graphitic interfaces by grafted molecular motifs, we now turn to the evaluation of a new molecule. We investigated vitamin B_2_ or riboflavin (RF) as a new RAO molecule for direct EMCC. The mechanism for the redox behavior of RF in dimethyl sulfoxide (DMSO) solvent was previously reported as a two-step reduction at fast scan rates with the formation of a semiquinone (RF˙^−^) at −1 V (*vs.* Fc/Fc^+^) followed by a second reduction of nitrogen in the isoalloxazine ring at −1.8 V (*vs.* Fc/Fc^+^) (RF^2−^) (Scheme 2a).^52–54^ At slower scan rates, a protonation reaction occurs between RF and RF˙^−^ species that produces RF_ox_^−^ and RFH˙ (Scheme S5), adding complexity to the CV response. However, preliminary electrochemical experiments were conducted using freely diffusing 1 mM RF in 0.1 M TBAPF_6_ MeCN (Fig. S8) to assess its potential to form a CO_2_ adduct. The cathodic sweep under argon contains a small redox peak at −1.22 V which we ascribe to the formation of RF˙^−^ and another larger peak at −2.14 V we attribute to the reduction of RF˙^−^, RF_ox_^−^, and RFH˙ (Scheme S5). Upon cycling under CO_2_ the peaks related to RF˙^−^ remain consistent, but a new peak emerges at −1.81 V and beyond that the current increases exponentially. Based on a change in the CV (Fig. S8) we posited that RF could bind CO_2_ in an analogous manner to AQ, but that surface confinement and alleviation of side reactions would greatly improve its CO_2_-binding performance. This is because the RF^2−^ species, or its intermediates, should be reactive and capable of binding to CO_2_ at either the nitrogen or oxygen site (Scheme 2b). There is the possibility of CO_2_ binding to both sites forming RF(CO_2_)_2_^2−^, but we believe steric hindrance would limit the occurrence of that species.^61^ Based on the attained spectra it is still difficult to assign where the binding of CO_2_ occurs due to spectral interference from CO_2_RR (Fig. S8).

### 3.5. Synthesizing and electrografting of the AMFD on graphene-on-gold

We immobilized RF to a graphene–on–gold interface. To this end, a riboflavin derivative, 10-(2-ammoniumethyl)-3-methyl flavin derivative (AMFD), was synthesized with an ethyl-ammonium chloride group as the R group (Scheme S1) such that a diazonium group could then be generated through the reaction with *t*-butyl nitrite for easy electrografting onto the electrode surface.^25,39–44^ However, the *t*-butyl nitrite reacts with amines, which may decompose RF. To prevent any degradation, the imide group in the ring (between the two carbonyls) was methylated. Inadvertently, the methylation of this imide inhibits proton transfers within the ring further limiting side reactions from occurring and potentially improving the ability of the RF core to bind CO_2_.^52^

**Fig. 4:**
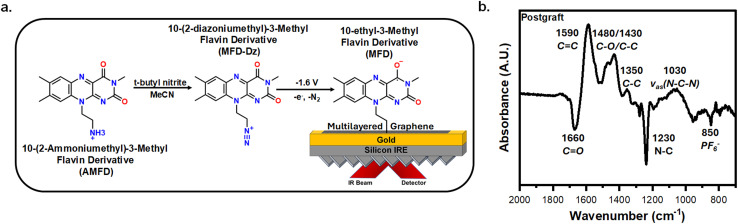
Electrografting of MFD onto graphene-on-gold. (a) The synthetic route for converting the AMFD to MFD-Dz followed by electrografting of onto a graphene-on-gold substrate. (b) A labelled spectra detailing the peaks observed after the graft. The reference of the spectra was taken at 0.4 V in 0.1 M TBAPF_6_ in MeCN with 1 mM MFD-Dz.

Fig. 4a portrays the reaction scheme for generating and electrografting a 10-(2-diazoniumethyl)-3-methyl flavin derivative (MFD-Dz) onto a graphene-on-gold electrode. The AMFD was converted into MFD-Dz following the same procedure for preparing AQ-Dz.^25,39–44^ To initiate the grafting of the MFD-Dz, a potential of −1.6 V was held for 10 minutes with the reference spectra was acquired by holding 0.4 V in 0.1 M TBAPF_6_ MeCN with 1 mM MFD-Dz. The *in situ* monitoring of the electrografting of the MFD-Dz displays a clear growth of both positive and negative peaks throughout the grafting process (Fig. S9). Peak assignments for MFD were determined from previously reported spectroscopic studies on RF (Table S1).^67–75^ The main peaks observed are the negatively growing 1660 cm^−1^ C=O stretch and N–C peak at 1230 cm^−1^, and the positively growing 1590 cm^−1^ C=C band, 1480/1430 cm^−1^ C–O/C–C stretches, 1350 cm^−1^ C–C stretch, and the asymmetrical bending of the isoalloxazine ring at 1030 cm^−1^ (Fig. 4b). The peaks observed correlate well with previously seen stretches of the RF precursor (Fig. S8).^67–75^

### 3.6. Characterizing the redox behavior of MFD-modified graphene-on-gold under argon and CO_2_

**Fig. 5:**
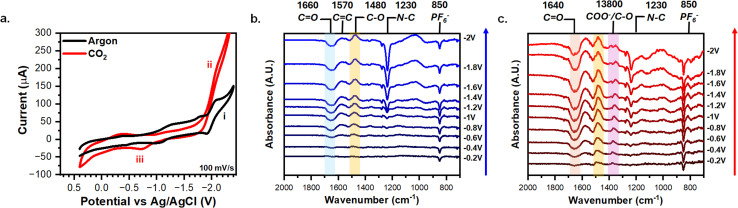
CVs and ATR-SEIRAS characterization of MFD-modified graphene-on-gold electrodes in 0.1 M TBAPF_6_ MeCN. (a) CVs of MFD-modified graphene-on-gold under argon or CO_2_ purge. Potential-dependent spectra MFD-modified graphene-on-gold under (b) argon or (c) CO_2_ purge with key peak highlighted and labeled. The reference spectra were taken at 0.4 V under either argon or CO_2_ saturation.

To verify the electrografting, the MFD-modified graphene-on-gold electrode was rinsed thoroughly and cycled in blank 0.1 M TBAPF_6_ in MeCN under argon and CO_2_ (Fig. 5a). The CV shows the reduction of the MFD with a main peak (i) at −1.99 V and 0.15 V peak-to-peak separation. This redox peak stems from surface-bound species as the cathodic and anodic peak current values scale linearly with increasing scan rates (Fig. S10). The average surface coverage obtained was 0.8 ± 0.2 nmol cm^−2^. Upon purging CO_2_, the CV shows a distortion of the original peak (ii) showing an increasing current at negative potentials, like that already described as originating from the CO_2_RR. Upon reversal, an oxidation peak (iii) appears at −0.74 V, presumably corresponding to the oxidation of the formed MFD-CO_2_ adducts (Fig. 5a).

Fig. 5b shows the potential-dependent spectra of MFD-modified graphene-on-gold under argon purge. At progressively more negative potentials the growing peaks are the 1570 cm^−1^ C=C stretch, 1480 cm^−1^ C–O stretch (yellow shaded), 1350 C–C stretch, and the 1030 cm^−1^ N–C–N band.^67–75^ The 1660 cm^−1^ C=O (blue shaded) and 1230 cm^−1^ N–C stretches both go negative at −1.2 V presumably caused by the formation of MFD˙^−^.^67–75^ The other peaks grow in intensity presumably due to conformation changes of the bound-MFD to alleviate electrostatic repulsion from applying negative potential at the electrode interface.^68^ Under CO_2_ the changes are not as pronounced until at −1.8 V where the CO_2_RR is observed with growth of 1640 cm^−1^ and 1380 cm^−1^ peaks (Fig. 5c). Additionally, continuously holding −1.5 V under CO_2_ purge does not generate any changes in the spectra, indicating that MFD˙^−^ species do not bind CO_2_ (Fig. S11).

### 3.7. Monitoring the capture and release of CO_2_ using MFD-modified graphene-on-gold

**Fig. 6:**
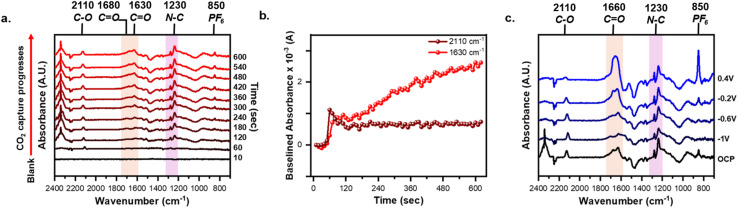
Time-resolved spectra of CO_2_ capture and release using MFD-modified graphene-on-gold with key peak highlighted and labeled. (a) *In situ* monitoring of the CO_2_ capture by holding −2.1 V for 1 min under argon purge, then 9 min of ambient CO_2_ capture without any applied potential. (b) Tracking the baselined absorbance value of the 2110 cm^−1^ and 1630 cm^−1^ peak during CO_2_ capture. The spectra at 10 s were used as the baseline. (c) The release of the CO_2_ is observed when holding progressively positive potentials for 10 min each under argon purge. The reference spectra was taken at −2.1 V under argon purge.

*In situ* monitoring of the CO_2_ capture using an MFD-modified electrode was performed following the same procedure used for ambient capture for AQ (Section 3.3), where bound MFD in the reduced form was allowed to interact with CO_2_ at open circuit. The reference spectra was the MFD-modified electrode held at −2.1 V under argon to form MFD^2−^ species at the interface. The spectral response from CO_2_ capture using MFD-modified electrodes differs from AQ-modified electrodes (Fig. 6a). The rising peaks are the 2110 cm^−1^ CO band, a broad peak from 1680 cm^−1^ to 1630 cm^−1^ (red shaded), the 1570 cm^−1^ C=C band, the 1520 cm^−1^ C=N band, the 1280 cm^−1^ C–C stretch, and the 1230 cm^−1^ C–N stretch (purple shaded). The peaks at 1680 cm^−1^ and 1630 cm^−1^ relate to C=O stretches which indicate the binding of CO_2_ to MFD. The C–O stretch at 2110 cm^−1^ again is observed possibly indicating the presence of carbon monoxide or bound-CO_2_ at the interface as previously observed with AQ-modified electrodes.^31,61^ The tracked peak heights at 2110 cm^−1^ and 1630 cm^−1^ show that the 2110 cm^−1^ peak spikes as soon as CO_2_ is introduced to the cell (∼60 s) but quickly plateau while the 1630 cm^−1^ peak continuously grows over 10 min (Fig. 6b). Although it is difficult to point the binding site, these spectral changes strongly suggest binding of CO_2_ to MFD.

To release the bound CO_2_, the MFD-modified electrode was subsequently held at −1 V, −0.6 V, −0.2 V, and 0.4 V for 10 min each under argon purge (Fig. S12). Again, these potentials align with distinct features in the CV shown in Fig. 5a, with potentials more negative than −0.74 V providing conditions for the MFD-(CO_2_) adduct to be present at the electrode surface, and with more positive potentials expected to release CO_2_. Fig. 6c compares the spectra after 10 minutes of each release potential. At −1 V the 1680 cm^−1^ peak shifts to 1705 cm^−1^ while the 1570 cm^−1^ grows and overlaps with 1630 cm^−1^ peak. At −0.6 V a broad peak forms from 1710 cm^−1^ to 1550 cm^−1^ centered at 1630 cm^−1^. At 0.2 V a broad peak grows with doublet peaks at 1680 cm^−1^ and 1630 cm^−1^. At 0.4 V the 2110 cm^−1^ shrinks significantly, a broad peak is observed centered at 1660 cm^−1^ corresponding to regeneration of MFD. Moreover, at 0.4 V the 850 cm^−1^ PF_6_^−^ peak spikes in intensity, once more suggesting a dramatic change in the charge of the electrode. Unlike the AQ-modified electrodes, the MFD-(CO_2_) species begins desorption of CO_2_ as early as −0.6 V as evidenced by the progressive modification of the broad peak between 1600–1700 cm^−1^, which culminates in the appearance of the quinone feature at 1660 cm^−1^ characteristic of the fully oxidized MFD species (Fig. 4b). As in the case of AQ, larger overpotentials lead to a higher degree of regeneration of the original redox form, although MFD shows spectroscopic activity significantly earlier than AQ (*i.e.*, at a more positive potential of ∼ −0.6 V for MFD compared to 0.4 V for AQ). These spectra demonstrate the viability of MFD to effectively electrochemically capture and release CO_2_ and the ability of our platform to screen novel RAO capture agents.

### 3.8. Comparing the carbon capture performances of AQ-modified and MFD-modified porous carbon electrodes

**Fig. 7:**
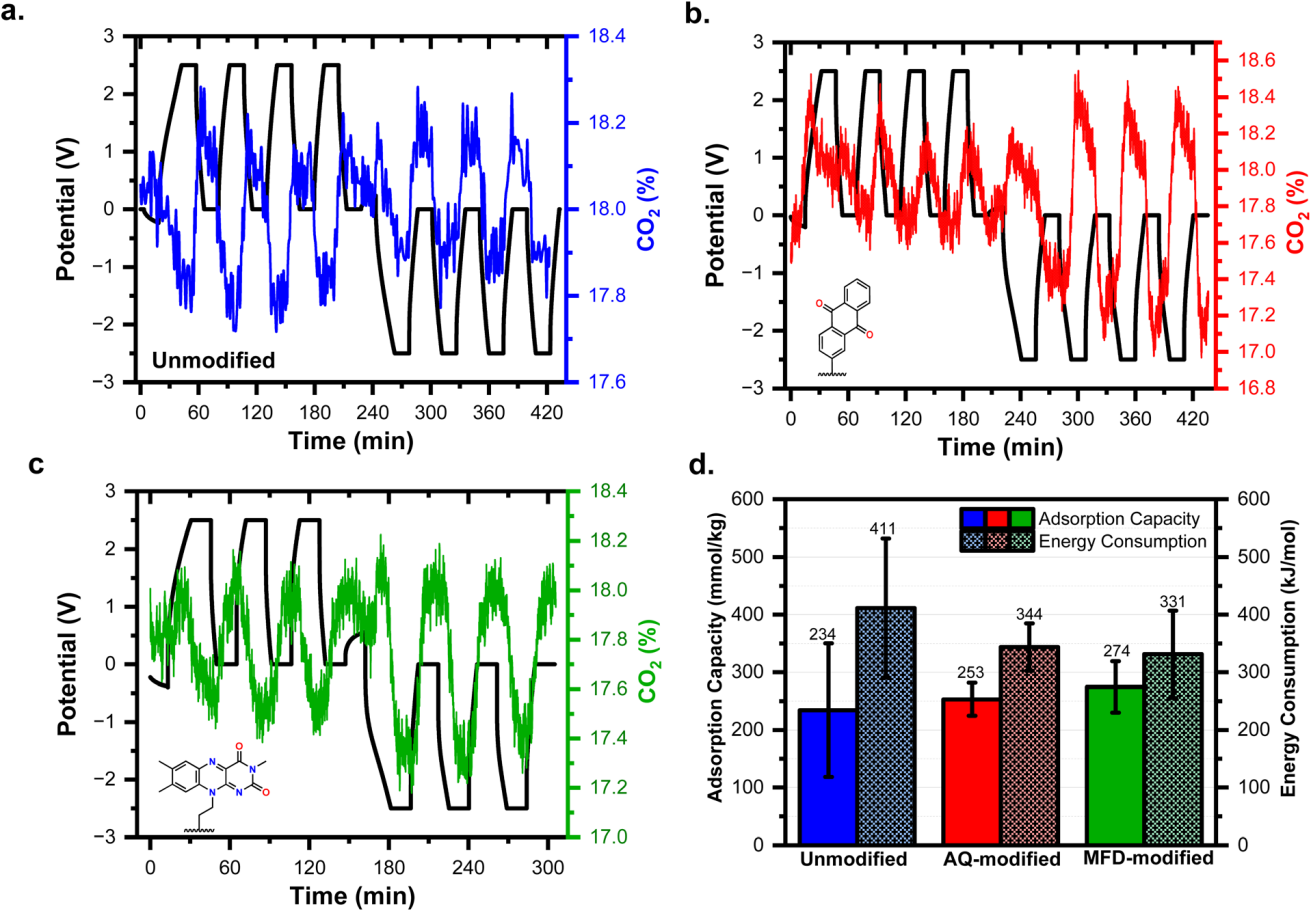
Monitoring the fluctuations in CO_2_% in a two-electrode flow cell under galvanostatic cycling using (a) a symmetric configuration with two YP-50 electrodes, (b) asymmetric configuration using a YP-50 electrode and an AQ-modified YP-50 electrode, and (c) asymmetric configuration using a YP-50 electrode and a MFD-modified YP-50 electrode, all in 0.5 M LiTFSI in PC as electrolyte. (d) The CO_2_ capture performance metrics of all three cell types tested.

While spectroscopic data show the possibility of CO_2_ adduct formation at the surface of the modified graphitic electrodes, it is relevant to evaluate the degree to which such binding events may remove gaseous CO_2_ in a prototype device. Fig. 7 displays results of preliminary electrochemical CO_2_ adsorption experiments using AQ- and MFD-modified YP-50 activated carbon electrodes that track the change in CO_2_ concentration using a custom electrochemical flow cell (Scheme S2). Modification of the YP-50 electrodes was carried out by scaling the diazonium grafting procedures described for graphene-on-gold electrodes (SI Section 2). The electrodes were first cycled using galvanostatic charge/discharge at 50 mA g^−1^ between 0 V and 2.5 V for three cycles and then between 0 V and −2.5 V for three cycles in 0.5 M LiTFSI in PC electrolyte using unmodified YP-50 as the counter electrode. Fig. 7a–c show the changes in cell potential and CO_2_ concentration. The total CO_2_ captured will have contributions from both the faradaic (*i.e.*, redox-triggered AQ or MFD capture) and non-faradaic double-layer charging (*i.e.*, supercapacitve swing adsorption).^25,76–78^ The double-layer contribution is observed *via* the oscillation of the CO_2_ with the cell contains two unmodified YP-50 electrodes (Fig. 7a and Scheme S3a). When the electrodes were charged to −2.5 V or 2.5 V, the CO_2_ analyzer detected a decrease in CO_2_%, indicating CO_2_ uptake by the electrochemical cell. Then, the CO_2_ was released when discharging to 0 V, as observed in the increases in CO_2_%. Fig. 7b–c show that the asymmetric electrode flow cells (Scheme S3b), where one YP-50 electrode was modified with AQ^2−^ or RF^2−^ species, have a larger overall decrease in CO_2_% than the symmetric YP-50 electrode flow cell. The CO_2_ adsorption capacity and energy consumption calculated from the data are reported in Fig. 7d, with the calculation details reported in the SI. The symmetric YP-50 electrode flow cell had the largest range in adsorption capacity and energy consumption due to the heterogeneous nature of CO_2_ capture using porous carbon electrodes.^78,79^ Conversely, the asymmetric electrode flow cells using AQ- and MFD-modified electrodes were more reproducible, with increased CO_2_ capture and decreased energetic requirements. Despite the MFD-containing flow cell operating more efficiently than the AQ-containing flow cell on average, all the results fall within standard error of each other. Further studies are needed to evaluate the performance of these systems over longer cycles to assess their viability and scalability. Regardless, these results imply that MFD-modified electrodes perform on par with AQ-modified electrodes and should be considered as a plausible alternative, and that both electrode surfaces are viable for electrochemical CO_2_ capture.

## 4. Conclusions

Herein we have successfully monitored the reversible electrochemical capture and release of CO_2_ from AQ- and MFD-modified graphitic electrodes using *in situ* ATR-SEIRAS. Our previously developed graphene-on-gold electrodes were modified with AQ and MFD through the generation and electrografting of diazonium functional groups while tracking the functionalization in real-time using the ATR-SEIRAS. AQ- and MFD-modified electrodes were characterized under argon and CO_2_ atmospheres both under continuous polarization where CO_2_RR dominates, and at open circuit following the reduction of the electrode-bound molecular motifs where CO_2_ capture and release is the main process observed. CO_2_ capture events were revealed primarily through the growth of a broad spectral feature around ∼1710–1630 cm^−1^ and release events were observed through the regeneration of an intense carbonyl stretch at ∼1660 cm^−1^. Our experiments differentiated through the spectral response the intermediates produced *via* CO_2_RR and CO_2_ capture processes at the modified carbon interfaces. We showed how continuous polarization at negative electrodes favor the production of CO_3_^2−^ as a byproduct, overshadowing the CO_2_ capture process using molecular motifs. In contrast, reduction of AQ or MFD followed by introduction of CO_2_ in the electrolyte while the electrode rested at open circuit results in distinct spectral features consistent with the formation of a surface-bound adduct. We further showed that these features respond to the application of key electrode potentials corresponding to redox events in the CV denoting capture/release processes. Overall, our results highlight relevant considerations for performing CO_2_ capture studies under different polarization conditions.

The use of graphene-on-gold electrodes adds on the capability to track interfacial processes at relevant graphitic carbon surfaces, potentially to screen for novel molecular motifs bound on electrode surfaces and validating their reaction mechanisms. For example, RF was unequivocally shown as a viable CO_2_ capture agent, and although its spectral features were like those observed for AQ suggesting similar capture/release mechanisms, their potential responses were different, with MFD showing some advantage in terms of overpotential needed for the release process. The carbon capture performance of AQ- and MFD-modified porous carbon electrodes, assessed using a custom electrochemical flow cell, showed that MFD displays an improvement in capture capacity and energy consumption. Improvements to the surface modifications needed for device-level capture as informed by interfacial studies, and optimization to the loading and activity of bound redox motifs will be needed to make greater strides in our ability to efficiently capture CO_2_. Nonetheless, this work highlights relevant considerations for performing CO_2_ capture studies under polarization, illuminates key surface intermediates, and demonstrates the ability to screen novel RAO capture agents using ATR-SEIRAS on novel graphene-on-gold electrodes.

## Author contributions

A. S. contributed to conceptualization, investigation, methodology, formal analysis, validation, visualization, data curation, writing the original draft, reviewing, and editing. J. R. contributed to conceptualization, investigation, methodology, validation, visualization, writing the original draft, reviewing, and editing. J. N. contributed to conceptualization, investigation, methodology, validation, reviewing, and editing. A. F. contributed to investigation, formal analysis, validation, visualization, and writing the original draft, reviewing, and editing. K. M contributed to investigation, validation, and reviewing and editing. S. T. P. contributed to investigation, validation, and reviewing and editing of the manuscript revisions. R. B. contributed to resources, supervision, reviewing, and editing. J. M. D contributed to supervision, reviewing, and editing. S. C. Z contributed to funding acquisition, resources, supervision, reviewing, and editing. V. A. contributed to funding acquisition, resources, supervision, formal analysis, reviewing and editing. J. R. L contributed to funding acquisition, resources, supervision, project administration, reviewing and editing.

## Conflicts of interest

There are no conflicts to declare.

## Supplementary Material

SC-016-D5SC05427C-s001

SC-016-D5SC05427C-s002

## Data Availability

The data supporting this article have been included as part of the supplementary information (SI). Supplementary information: the synthetic procedures for synthesizing AMFD, YP50 electrode fabrication and functionalization procedures, and flow cell setup, are all in the supplementary information. Additional supplementary figures or schemes are mentioned in the main text, additional experimental details or equations used. See DOI: https://doi.org/10.1039/d5sc05427c.
