# Supplementary material for: Real-time monitoring of the reversible capture and release of CO2 on anthraquinone and riboflavin-modified graphitic electrodes using ATR-SEIRAS
Source: Chem Sci. 2025 Nov 4;16(48):23189–202. doi: 10.1039/d5sc05427c (PMC12584010; doi:10.1039/d5sc05427c)
Supplement: SC-016-D5SC05427C-s002 [file SC-016-D5SC05427C-s002.pdf]

Supplementary Information (SI) for Chemical Science.
This journal is © The Royal Society of Chemistry 2025

Supplementary Information

Real-time Monitoring of the Reversible Capture and Release of CO_2 on Anthraquinone and Riboflavin-Modified Graphitic Electrodes using ATR-SEIRAS

Abdur-Rahman Siddiqui [a±], Joel Roberts [a±], Jeanne N'Diaye [a,b±], Alan L. Ferris [±d], Kristin Martin [a], Seth T. Putnam [a], Rohit Bhargava [b,c], Jahan Dawlaty [e], Steven C. Zimmerman [a, f], Veronica Augustyn [d], and Joaquín Rodríguez-López [a,b*]

[a] Department of Chemistry, University of Illinois Urbana-Champaign, Urbana, Illinois, 61801, *United States*

[b] The Beckman Institute for Advanced Science and Technology, University of Illinois Urbana-Champaign, Urbana, Illinois 61801, *United States*

[c] Department of Bioengineering, University of Illinois Urbana-Champaign, Urbana, Illinois 61801, *United States*

[d] Department of Materials Science and Engineering, North Carolina State University, Raleigh, North Carolina, 27695, *United States*

[e] Department of Chemistry, University of Southern California, Los Angeles, California 90007, *United States*

[f] Department of Chemistry, Ben-Gurion University of the Negev, Beer Sheva, 8410501, *Israel*

[±]: The authors contributed equally

Contents

SI.1. Synthetic Procedures:......5

Scheme S1: Synthetic route from riboflavin to 10-(2-ammoniumethyl)-3-methyl flavin derivative (AMFD). 5

Compound 1 5

Compound 2 5

Compound 3 6

10-(2-Ammoniumethyl)-3-Methyl Flavin Derivative (AMFD) 6

SI.2. YP-50 Electrode Preparation, Functionalization, and Flow Cell Setup for Tracking CO_2 Adsorption......7

YP-50 Electrode Preparation and Functionalization:......7

Flow Cell Setup for Tracking CO_2 Adsorption: 7

Scheme S2: The schematic of the custom flow cell device. 7

Scheme 3: **(a)** The symmetric configuration with two YP-50 electrodes. **(b)** The asymmetric configuration using a YP-50 electrode and an AQ-modified or MFD-modified YP-50 electrode......8

SI.3. Supplementary Figures, Schemes, and Tables: 9

Table S1: IR Assignments of AQ, $AQ^{\bullet -}$, AQ^{2-}, AQ-CO_2, RF/MFD, MFD-CO_2, CO_2, CO_3^{2-}, PF_6^-......9

Figure S1: **(a)** Time-resolved spectra taken during the electrografting of AQ-Dz. **(b)** Final spectra taken of the electrografting of AQ-dz onto a graphene-on-gold electrode performed at varying potentials in 0.1 M $TBAPF_6$ in MeCN. The background spectra were taken at 0.4 V under argon. 10

Scheme S4: Mechanistic depictions of the electrochemical reduction of AQ and subsequent binding to CO_2 through the ECEC reaction mechanisms. 10

Figure S2: **(a)** CVs of the AQ-modified graphene-gold at different scan rates in argon purged blank 0.1 M $TBAPF_6$ MeCN solution. **(b)** A plot of the peak current at both redox peaks for AQ vs. scan rate......11

Figure S3: (a)Time-resolved spectra of the CO2RR using unmodified graphene-gold with key peaks highlighted and labeled. For the CO2RR, -2.1 V was held for 10 min under CO2 purge. (b) Tracking the baselined height of the 1640 cm-1 peak for the duration of CO2RR. (c) CVs of unmodified graphene-gold in 0.1M TBAPF6 in MeCN under argon or CO2 purge. The background of the spectra was taken at -2.1 V under argon......11

Figure S4: Time-resolved spectra of attempting CO_2 capture by holding -1.5 V for 10 min using an AQ-modified electrode under CO_2 saturation. 12

Figure S5: Spectra of the AQ-modified graphene-gold under CO_2 purge while holding -2.1 V and immediately after when no potential is applied. The background of the spectra was taken at -2.1 V under argon. 12

Figure S6: Time-resolved spectra of holding -2.1 V for 1 min then removing the applied potential and purging with CO_2 with an unmodified graphene-gold electrode. 13

Figure S7: Time-resolve spectra of applying different potentials to release CO_2 bound at AQ-modified electrodes in an argon purge. The potentials were **(a)** -1 V, **(b)** -0.6 V, **(c)** -0.2 V, **(d)** 0.4 V. 13

Figure S8: CVs and EC-SEIRAS characterization of freely diffusing RF in 0.1M TBAPF6 MeCN. **(a)** CV 1mM RF in 0.1M TBAPF6 MeCN using a graphene-on-gold electrode under argon and CO2. **(b)** CV of 1 mM RF under CO2 at different scan rates (25-500 mV/s). Potential-dependent spectra of freely diffusing RF under **(c)** argon and **(d)** CO_2 purge. The background spectra were taken at 0.4 V. 14

Scheme S5: Depictions of various side reactions that occur when reducing RF in non-aqueous solvents. 15

Figure S9: **(a)** Time-resolved spectra taken during the electrografting of MFD-Dz. **(b)** Final spectra taken of the electrografting of MFD-dz onto a graphene-on-gold electrode performed at varying potentials in 0.1 M $TBAPF_6$ in MeCN. The background spectra were taken at 0.4 V under argon. 16

Figure S10: **(a)** CVs of the MFD-modified graphene-gold at different scan rates in argon purged blank 0.1 M $TBAPF_6$ MeCN solution. A plot of the peak current at both redox peaks for AQ vs. **(b)** scan rate and **(c)** the square-root of the scan rate. 16

Figure S11: Time-resolved spectra of attempting a CO2 capture by holding -1.5 V for 10 min using a MFD-modified electrode. 17

Figure S12: Time-resolve spectra of applying different releasing potentials to CO_2-bound MFD-modified electrodes under argon purge. The releasing potentials used are **(a)** -1 V, **(b)** -0.6 V, **(c)** -0.2 V, **(d)** 0.4 V. 17

Figure S13: Cyclic voltammetry of 2-electrode flow cell with expanding voltage window from 0.5 V to 2.5 V, both positive and negative for **(a)** AQ-functionalized and **(b)** MFD-functionalized electrodes. 18

Figure S14: Spectra of **(a)** AQ-modified and **(b)** MFD-modified electrodes after ambient capture, after releasing CO_2 by applying 0.4 V, and applying -2.1 V after release. 18

Figure S15: **(a)** Time-resolve spectra were taken at OCP immediately after CO2RR using an unmodified electrode and **(b)** tracking the peak height of the 1640 cm-1 peak for the duration of CO2RR. The background of the spectra was taken at -2.1 V under argon. 19

Figure S16: **(a)** Time-resolve spectra were taken at OCP for 10 min after ambient capture using an **(a)** AQ-modified electrode and **(b)** MFD-modified electrode. Tracking the peak height of the 1630 cm^{-1} during the OCP for **(c)** AQ-modified electrode and **(d)** MFD-modified electrode. The background of the spectra was taken at -2.1 V under argon. 19

Figure S17: CVs and EC-SEIRAS characterization of freely diffusing 2-aminoanthraquinone (2-AAQ) using a graphene-on-gold electrodes in 0.1M $TBAPF_6$ MeCN. **(a)** CVs of 2-AAQ in

a 0.1M $TBAPF_6$ MeCN under argon and CO_2 purge. Potential-dependent spectra 2-AAQ under **(b)** argon or **(c)** CO_2 purge. The background spectra were taken at 0.4 V under argon and CO_2 separately. 20

Figure S18: CVs and EC-SEIRAS characterization of freely diffusing AMFD using graphene-on-gold electrodes in 0.1M $TBAPF_6$ MeCN. **(a)** CVs of AMFD in a 0.1M $TBAPF_6$ MeCN under argon and CO_2 purge. Potential-dependent spectra AMFD under **(b)** argon or **(c)** CO_2 purge. The background spectra were taken at 0.4 V under argon and CO_2 separately. 20

Figure S19: Potential-dependent spectra unmodified graphene-gold under **(b)** argon or **(c)** CO_2 purge. The background spectra were taken at 0.4 V under argon and CO_2 separately......20

Figure S20: Potential-dependent spectra for **(a)** AQ- and **(b)** MFD-modified graphene-gold under argon in 0.1 M $TBAPF_6$ in MeCN first dried over activated molecular sieves (4 angstrom) followed by fractional distillation from calcium hydride at a high reflux ratio under an argon atmosphere. 21

Figure S21: Time-resolve spectra of **(a)** CO_2RR with an unmodified graphene-gold and **(b)** ambient capture of CO_2 with a MFD-modified graphene-on-gold under CO_2 atmosphere in 0.1 M $TBAPF_6$ in MeCN first dried over activated molecular sieves (4 angstrom) followed by fractional distillation from calcium hydride at a high reflux ratio under an argon atmosphere. 21

Figure S22: Expanded spectrum from **(a)** Figure 3a and **(b)** Figure S3a to show the region 4000-700 cm^{-1}. 22

SI.4. CO_2 capture calculations 22

Figure S23: Representative CO_2 concentration profile during the electrochemical flow cell CO_2 capture step from $t0$ to tf, demonstrating the integration method used to calculate the volume of CO_2 removed from the gas stream. The shaded region represents the integrated difference between the baseline and the instantaneous concentration, quantified using Equation 2. 24

SI.1. Synthetic Procedures:

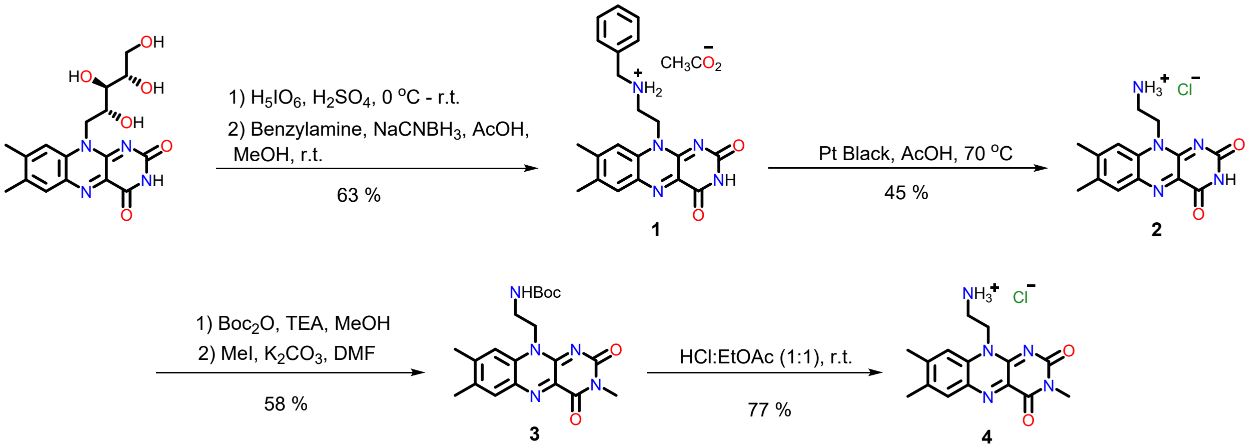

Scheme S1: Synthetic route from riboflavin to 10-(2-ammoniumethyl)-3-methyl flavin derivative (AMFD).

Compound 1

In a 500 mL round bottom flask, riboflavin (4.00 g, 10.6 mmol) was suspended in 140 mL of 1 M H_2SO_4, and the suspension was cooled with an ice bath. 50 mL of aqueous periodic acid (9.21 g, 40.4 mmol). The mixture was stirred at ice-bath temperature for 30 min and then allowed to warm to room temperature and allowed to stir with the avoidance of light. After, it was confirmed that the reaction mixture became a clear red-orange solution. A few drops of 1-octanol were added to the solution, and the pH of the solution was adjusted to 4.0 by addition of solid sodium bicarbonate. After the orange solid formed, it was filtered and washed with cold water (3x), cold methanol (3x) and ether (3x) then dried in over vacuum at room temperature to afford the crude orange solid. The crude was suspended in the mixture of 70 mL of methanol and 1 mL of acetic acid. Benzylamine (5.84 g, 5.95 mL g, 54.5 mmol) and sodium cyanoborohydride (0.984 g, 15.7 mmol) were added dropwise to the suspension. The reaction mixture was stirred at room temperature for 15 h with avoidance of light. The yellow solid was filtered then rinsed with cold methanol (3x), with ether (3x), and then dried in vacuo at room temperature to afford 2.91 g (63 % yield) of the yellow-orange solid. ^{1}H NMR (500 MHz, DMSO) δ 11.30 (s, 1H), 7.87 (s, 1H), 7.80 (s, 1H), 7.31 – 7.23 (m, 4H), 7.19 (t, J = 7.0 Hz, 1H), 4.69 (t, J = 7.0 Hz, 2H), 3.77 (s, 2H), 2.91 (t, J = 6.7 Hz, 2H), 2.48 (s, 3H), 2.39 (s, 3H), 1.91 (d, J = 2.5 Hz, 2H). ^{13}C NMR (151 MHz, DMSO) δ 172.48, 160.45, 156.08, 150.78, 146.72, 137.58, 136.15, 134.29, 131.71, 131.36, 128.52, 128.36, 127.04, 116.92, 53.00, 45.58, 40.54, 21.54, 21.08, 19.24. HR-ESI-MS: m/z calc. for $C_{21}H_{22}N_5O_2^+$ (M - $C_2H_3O_2^-$): 376.18; found: 376.18.

Compound 2

To a 250 mL round bottom flask, compound **1** (2.40 g, 5.51 mmol) and platinum black (222 mg) was added and dissolved in 100 mL of acetic acid. With a condenser attached, the system was replaced with a nitrogen atmosphere and then with a hydrogen atmosphere via a balloon filled with hydrogen gas. The mixture was stirred at 70 ˚C for 15 h with avoidance of light. After cooling, the reaction mixture was diluted with 100 mL of methanol, and insoluble materials were removed by filtration. The solution was further diluted with 100 mL of methanol with the subsequent addition of 55 mL of 1:1 conc. HCl / EtOAc which was allowed to stir for 30 min with the avoidance of

light. The resultant the dark yellow precipitate was resuspended in 30 mL of ethanol and vigorously stirred in a water bath at 60 ˚C for 3 h. After cooling, the solid was filtered, washed with cold ethanol (3x) and dried in vacuo at room temperature to afford 0.800 g (45 % yield) of a dark yellow solid. ^{1}H NMR (600 MHz, DMSO) δ 11.42 (s, 1H), 8.04 (s, 3H), 7.95 (d, J = 1.1 Hz, 1H), 7.94 (s, 1H), 4.88 (t, J = 6.6 Hz, 2H), 3.21 (q, J = 6.1 Hz, 2H), 2.53 (s, 3H), 2.41 (d, J = 0.9 Hz, 3H). ^{13}C NMR (151 MHz, DMSO) δ 151.53, 147.41, 137.77, 134.37, 131.72, 131.07, 116.37, 41.75, 36.78, 21.04, 19.20. HR-ESI-MS: m/z calc. for $C_{14}H_{16}N_5O_2^+$ (M - Cl^-): 286.13; found: 286.13.

Compound 3

To the solution of compound **2** (0.51 g, 4.3 mmol) in 40 mL of MeOH was added di-tert-butyl dicarbonate (0.38 g, 1.7 mmol) and Et_3N (1.3 mL, 0.96 g, 9.5 mmol). The mixture was stirred for 3 h at room temperature with the avoidance of light, and then concentrated under reduced pressure. The residue was dissolved in $CHCl_3$ (100 mL) and washed sequentially with 30 mL of water (3x), 30 mL of 2N HCl (3x), and 30 mL of brine. The organic phase was dried over $MgSO_4$ and filtered and concentrated under reduced pressure to give a crude orange solid. This solid, iodomethane (1.2 mL, 2.7 g, 19 mmol), and anhydrous K_2CO_3 (0.700 g, 5.1 mmol) in DMF (13 mL) was stirred for 8 h at room temperature with the avoidance of light at room temperature. The resulting reaction mixture was poured into 200 mL of water while stirring. The precipitate that formed was collected via filtration and dried under reduced pressure. The resulting crude product was purified by short column chromatography on silica gel (DCM:MeOH = 100 :1 - 20 : 1) to afford 0.370 g (58 % yield) a dark orange solid. ^{1}H NMR (600 MHz, $CDCl_3$) δ 8.03 (s, 1H), 7.88 (s, 1H), 4.84 (t, J = 6.9 Hz, 2H), 3.63 (q, J = 6.6 Hz, 2H), 3.50 (s, 3H), 2.56 (s, 3H), 2.44 (s, 3H), 1.41 (s, 9H). 13C NMR (151 MHz, CDCl3) δ 160.22, 156.58, 156.19, 148.87, 148.40, 136.97, 135.47, 135.15, 132.60, 131.67, 115.99, 80.23, 53.57, 44.24, 37.95, 28.88, 28.45, 21.80, 19.62. HR-ESI-MS: m/z calc. for $C_{20}H_{25}N_5O_4$: 400.19; found: 400.19.

10-(2-Ammoniumethyl)-3-Methyl Flavin Derivative (AMFD)

To a solution of compound **3** (0.500 g, 1.25 mmol) in $CHCl_3$ (24 mL) was added 4 N HCl/EtOAc (24 mL) dropwise manner. The resulting mixture was stirred overnight at room temperature with the avoidance of light. After addition of Et_2O (100 mL), the resulting precipitate was collected by filtration and washed with $CHCl_3$ and Et_2O to afford 321.80 mg (77 %) of a golden-yellow solid. ^{1}H NMR (600 MHz, DMSO) δ 8.07 (s, 3H), 8.00 (d, J = 1.1 Hz, 1H), 7.98 (s, 1H), 4.91 (t, J = 6.6 Hz, 2H), 3.30 (s, 3H), 3.21 (q, J = 6.2 Hz, 2H), 2.54 (s, 3H), 2.42 (d, J = 0.9 Hz, 3H). A ^{13}C NMR spectrum could not be measured due to the extremely low solubility of the title compound. HR-ESI-MS: m/z calc. $C_{15}H_{18}N_5O_2^+$: 300.15; found: 300.15

SI.2. YP-50 Electrode Preparation, Functionalization, and Flow Cell Setup for Tracking CO_2 Adsorption

YP-50 Electrode Preparation and Functionalization:

YP-50 carbon electrodes were fabricated with a 80:10:10 by weight ratio of activated carbon, acetylene black, and PTFE binder. Batches of electrode material were made in 200 mg increments. 160 mg of YP-50 powder and 20 mg of acetylene black were added to a 12 mL polypropylene container. 1 mL of ethanol was added to the powder mixture. The container was then placed into a Thinky Mixer to incorporate. The mixing parameters were 2000 rpm for 5 minutes for each cycle. 20 mg of PTFE binder was then added to the mixture to complete a mixing cycle. After mixing with the binder, the solution began to congeal into a dough-like consistency. The material was removed from the container and spread onto a 2 cm x 2 cm sheet of titanium mesh. Two pieces of wax paper were put on either side of the electrode and it was put through a calender until it reached a desired mass loading of 15-20 mg/cm^2. The excess material was then trimmed off the edges and the electrodes were dried at 60°C in air in a convection oven. Then the same electrodes were functionalized with AQ and MFD following the same diazonium generation procedures described below. The modified electrodes were then placed into the flow cell in which the CO_2 diffuses through the electrochemical cell. The electrodes were soaked in grafting solutions for 24 hours before polarizing at -1.6 V in 5mM AQ-Dz or MFD-Dz solution dissolved in 0.1 M $TBAPF_6$ in MeCN for 10 minutes. Electrodes were rinsed with blank MeCN and dried with argon before electrochemical measurements.

Flow Cell Setup for Tracking CO_2 Adsorption:

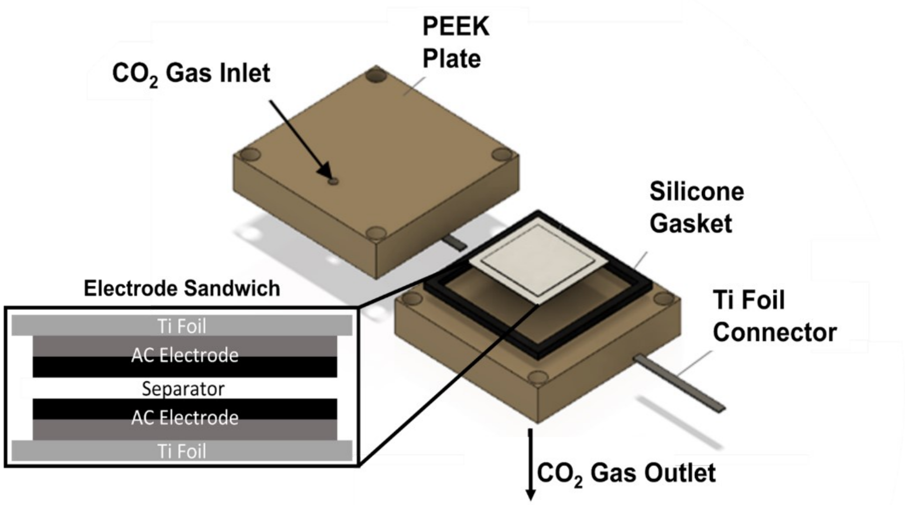

Scheme S2: The schematic of the custom flow cell device.

We designed a flow cell to measure the change in %CO_2 upon electrode polarization also known as supercapacitve swing adsorption[77-78]. Two polyether ether ketone (PEEK) plates were machined to house the electrochemical cell. The top plate included an inlet gas port with an internal gas diffusion pathway. A gas outlet was machined into the bottom plate. Gas flowed from the cylinder (80% argon, 20% CO_2; 99.9%, Arc3 Gases) into a mass flow controller (Alicat) set to a 5 sccm flow rate. The gas then passed through the flow cell and into a CO_2 analyzer (Model 906, Quantek Instruments). The analyzer reported an instantaneous concentration of CO_2 of the total flow in %CO_2. Baseline measurements were 17.87 ± 0.12% CO_2 and were gathered after a 30 minute normalization period where the concentration reached a consistent value. Variation in our baseline values in our testing occurred due to our differing background gas from the Quantek calibration mixture, which uses CO_2/N_2. However, the baseline values fell within the range expected when using argon as the balancing gas in our 20% CO_2 gas cylinder. Swagelok fittings were used at each gas inlet. Each electrode was saturated with 0.5 M LiTFSI in PC along with the separator for 30 minutes prior to assembly. Cell components were then placed in the center of the top base plate in the order of modified electrode, separator, counter electrode. Two titanium foil sections were placed under each electrode, which provided an electrical connection once the cell was sealed. A 3/16" fluorosilicone rubber gasket was then placed along the edge of the top plate and the bottom plate was screwed on, which sealed the system and applied pressure to the supercapacitor cell. A potentiostat (Biologic SP-50) was used to perform electrochemical experiments, including cyclic voltammetry (CV) and galvanostatic charge discharge (GCD).

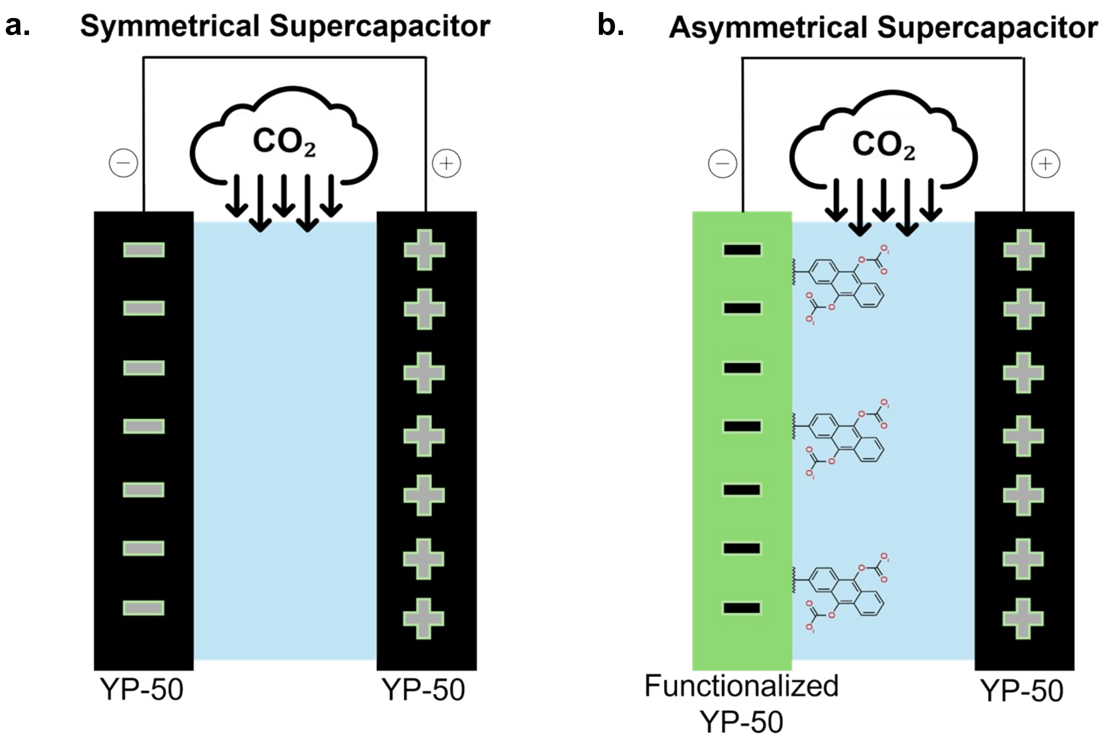

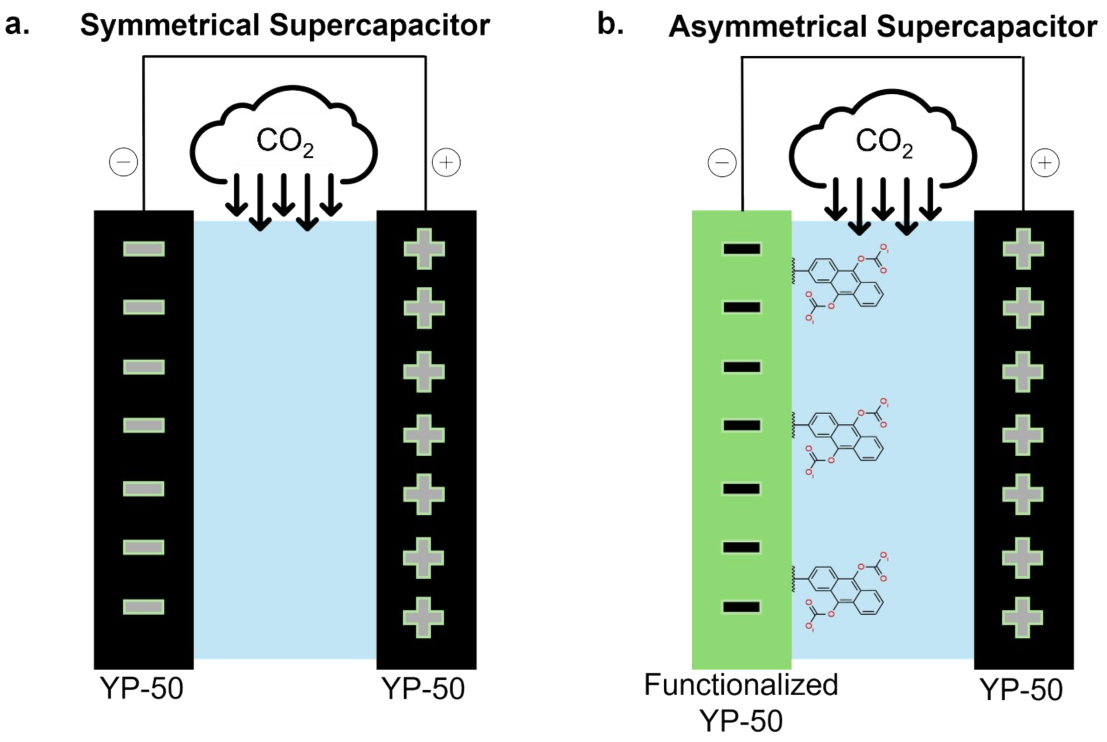

Scheme 3: (a) The symmetric configuration with two YP-50 electrodes. **(b)** The asymmetric configuration using a YP-50 electrode and an AQ-modified or MFD-modified YP-50 electrode.

SI.3. Supplementary Figures, Schemes, and Tables:

Table S1: IR Assignments of AQ, AQ$^{\bullet-}$, AQ^{2-}, AQ-CO_2, RF/MFD, MFD-CO_2, CO_2, CO_3^{2-}, PF_6^-.

Molecule	Wavenumber (cm^{-1})	IR Assignments
AQ	1740	C=O
	1660	C=O
	1570	C=C
	1270	C-C
	1230	C-C
AQ$^{\bullet-}$	1480	C-O
	1360	C-C
	1050	C-C
AQ^{2-}	1370	C-C
	1350	v(C-O)
AQ-CO_2	2110	CO
	1710-1600	C=O
	1600-1560	C=C
RF/MFD	1660	C=O
	1480	C-O
	1430	C-C
	1350	C-C
	1230	N-C
	1030	v(N-C-N)
MFD-CO_2	2110	CO
	1680	C=O
	1630	C=O
CO_2	2350	O=C=O
CO_3^{2-}	1640	C=O
	1380	COO^-
PF_6^-	850	PF_6^-

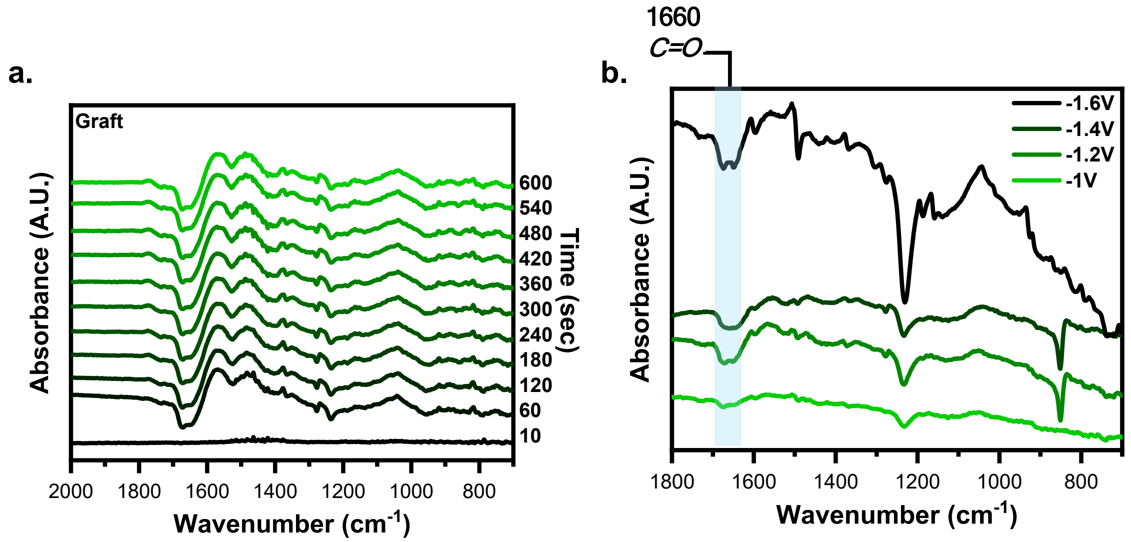

Figure S1: (a) Time-resolved spectra taken during the electrografting of AQ-Dz. **(b)** Final spectra taken of the electrografting of AQ-dz onto a graphene-on-gold electrode performed at varying potentials in 0.1 M $TBAPF_6$ in MeCN. The background spectra were taken at 0.4 V under argon.

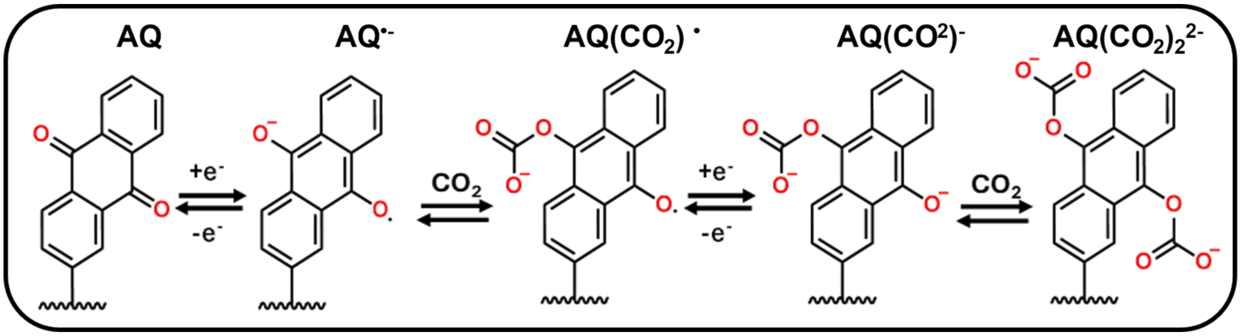

Scheme S4: Mechanistic depictions of the electrochemical reduction of AQ and subsequent binding to CO_2 through the ECEC reaction mechanisms.

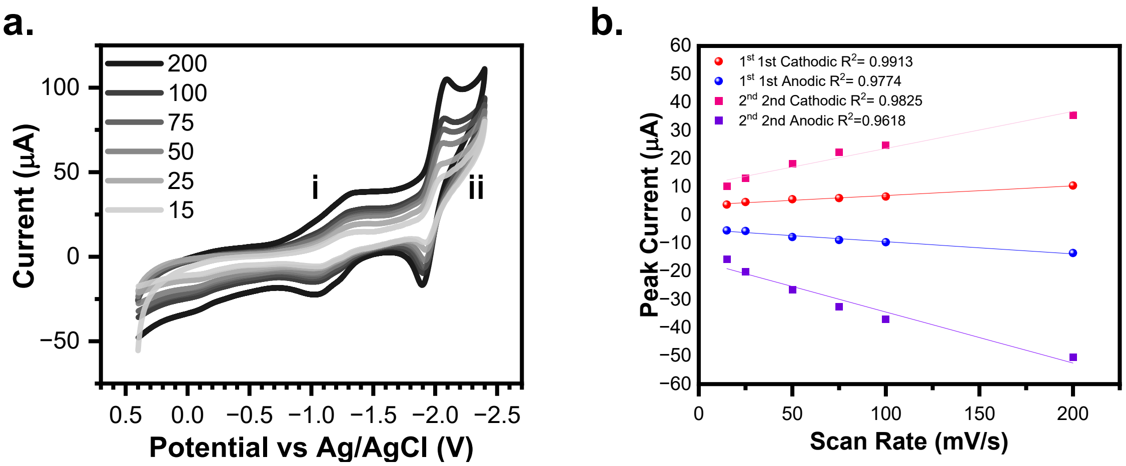

Figure S2: (a) CVs of the AQ-modified graphene-gold at different scan rates in argon purged blank 0.1 M $TBAPF_6$ MeCN solution. **(b)** A plot of the peak current at both redox peaks for AQ vs. scan rate.

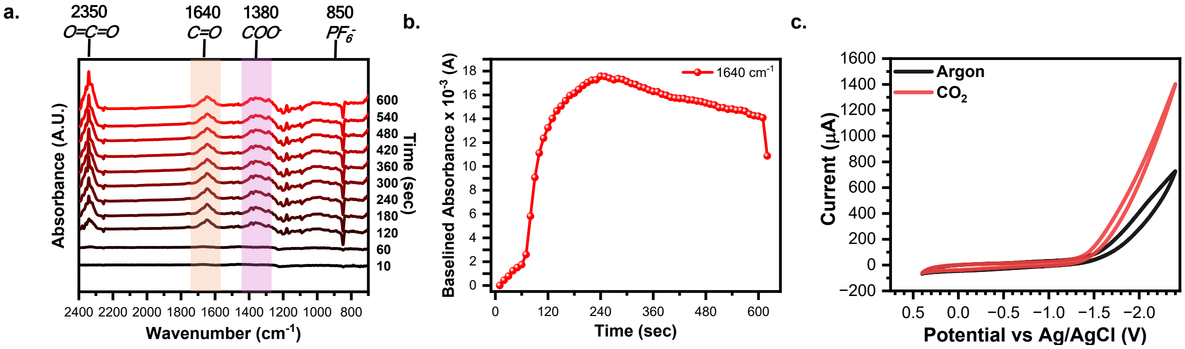

Figure S3: (a)Time-resolved spectra of the CO2RR using unmodified graphene-gold with key peaks highlighted and labeled. For the CO2RR, -2.1 V was held for 10 min under CO2 purge. (b) Tracking the baselined height of the 1640 cm-1 peak for the duration of CO2RR. (c) CVs of unmodified graphene-gold in 0.1M TBAPF6 in MeCN under argon or CO2 purge. The background of the spectra was taken at -2.1 V under argon.

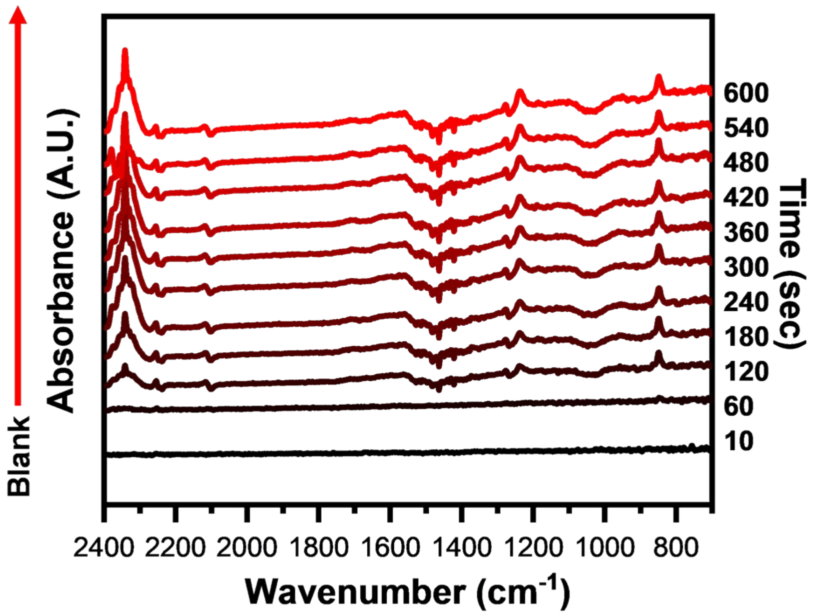

Figure S4: Time-resolved spectra of attempting CO_2 capture by holding -1.5 V for 10 min using an AQ-modified electrode under CO_2 saturation.

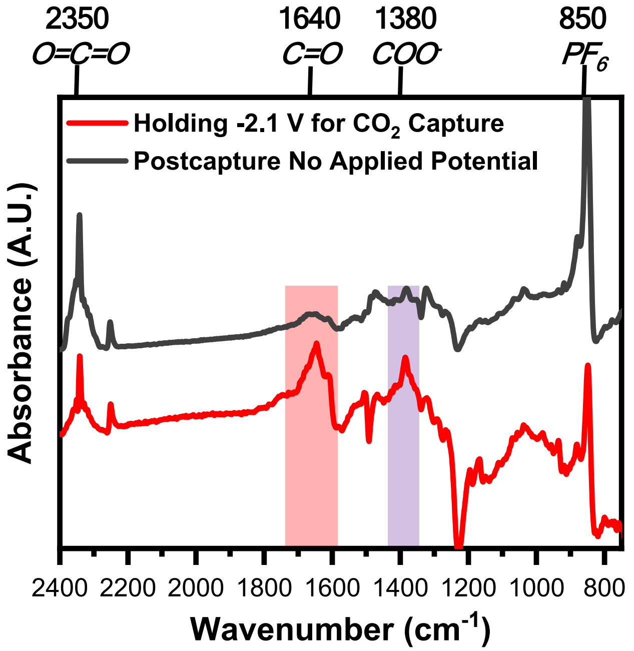

Figure S5: Spectra of the AQ-modified graphene-gold under CO_2 purge while holding -2.1 V and immediately after when no potential is applied. The background of the spectra was taken at -2.1 V under argon.

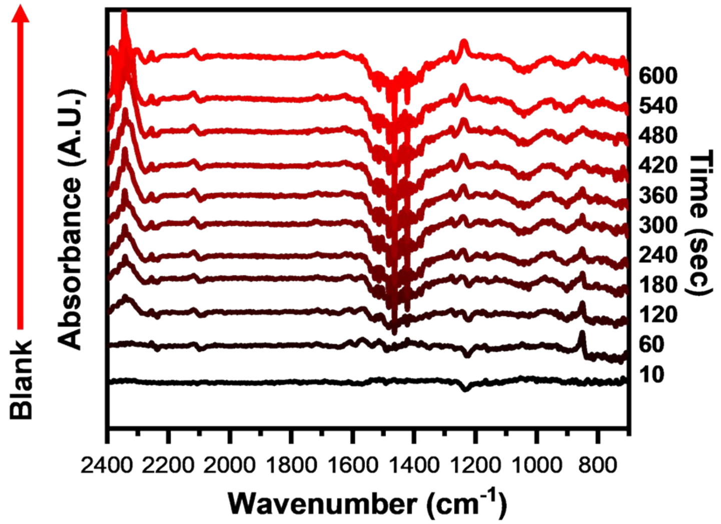

Figure S6: Time-resolved spectra of holding -2.1 V for 1 min then removing the applied potential and purging with CO_2 with an unmodified graphene-gold electrode.

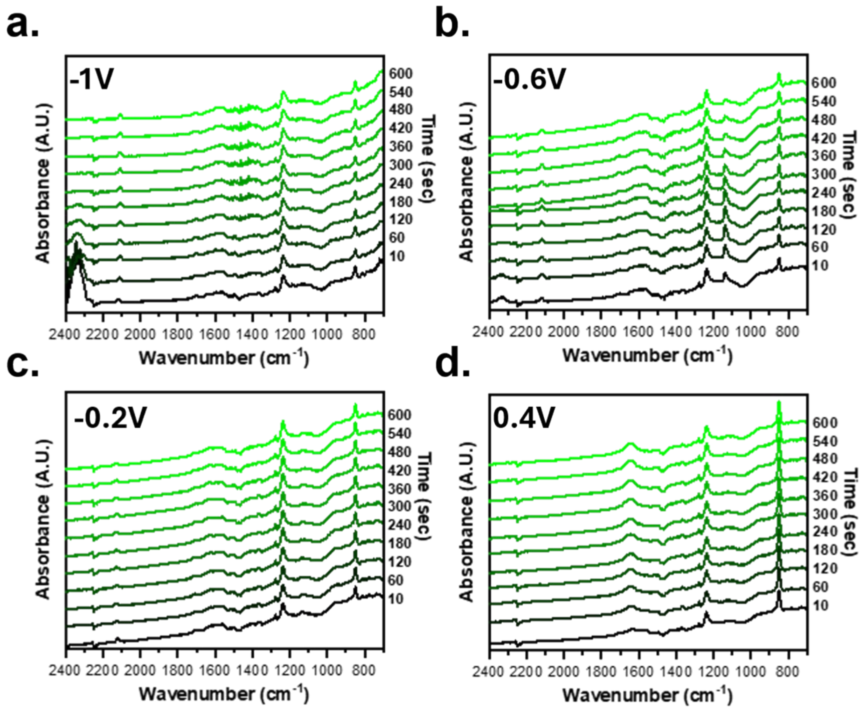

Figure S7: Time-resolve spectra of applying different potentials to release CO_2 bound at AQ-modified electrodes in an argon purge. The potentials were**(a)** -1 V, **(b)** -0.6 V, **(c)** -0.2 V, **(d)** 0.4 V.

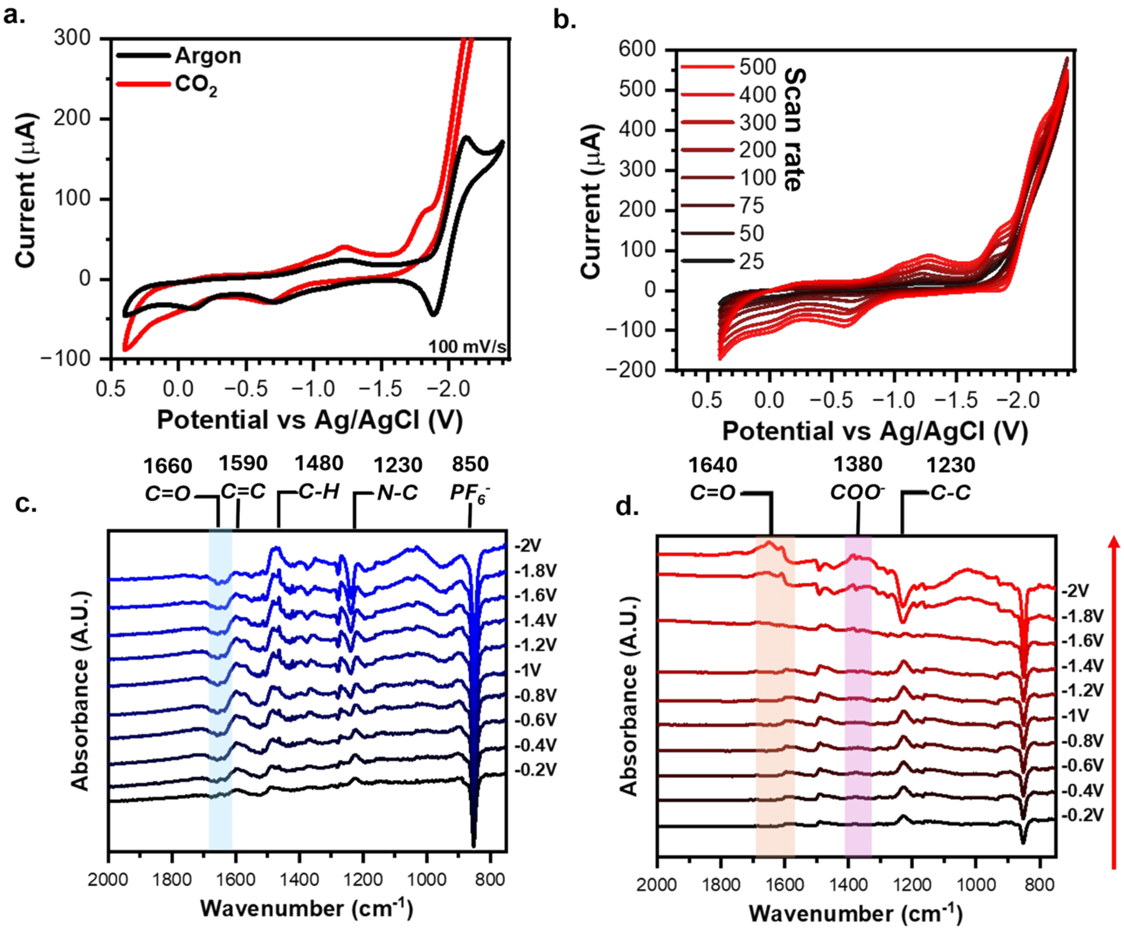

Figure S8: CVs and EC-SEIRAS characterization of freely diffusing RF in 0.1M TBAPF6 MeCN. **(a)** CV 1mM RF in 0.1M TBAPF6 MeCN using a graphene-on-gold electrode under argon and CO2. **(b)** CV of 1 mM RF under CO2 at different scan rates (25-500 mV/s). Potential-dependent spectra of freely diffusing RF under **(c)** argon and (**d)** CO_2 purge. The background spectra were taken at 0.4 V.

At slower scan rates, a protonation reaction occurs between RF and $RF^{\bullet -}$ species that produces RF_{ox}^{-} and $RFH^{\bullet}$ (**Scheme S5**), adding complexity to the CV response. However, preliminary electrochemical experiments were conducted using freely diffusing 1 mM RF in 0.1 M $TBAPF_6$ MeCN (**Figure S8**) to assess its potential to form a CO_2 adduct. The cathodic sweep under argon contains a small redox peak at -1.22 V which we ascribe to the formation of $RF^{\bullet -}$ and another larger peak at -2.14 V we attribute to the reduction of $RF^{\bullet -}$, RF_{ox}^{-}, and $RFH^{\bullet}$(**Scheme S5**). In the anodic sweep there is a peak at -1.88 V correlating to the oxidation of RF^{2-} and another peak at -0.71 V relating to the oxidation of $RF^{\bullet -}$ and any reduced side products (RF_{ox}^{2-}, RFH^{-}, **Scheme S5**).[29–31] The smaller anodic peak at -0.12 V could be due to further oxidation of RF_{ox}^{-}/$RFH^{\bullet}$ back to RF (**Scheme S5**).

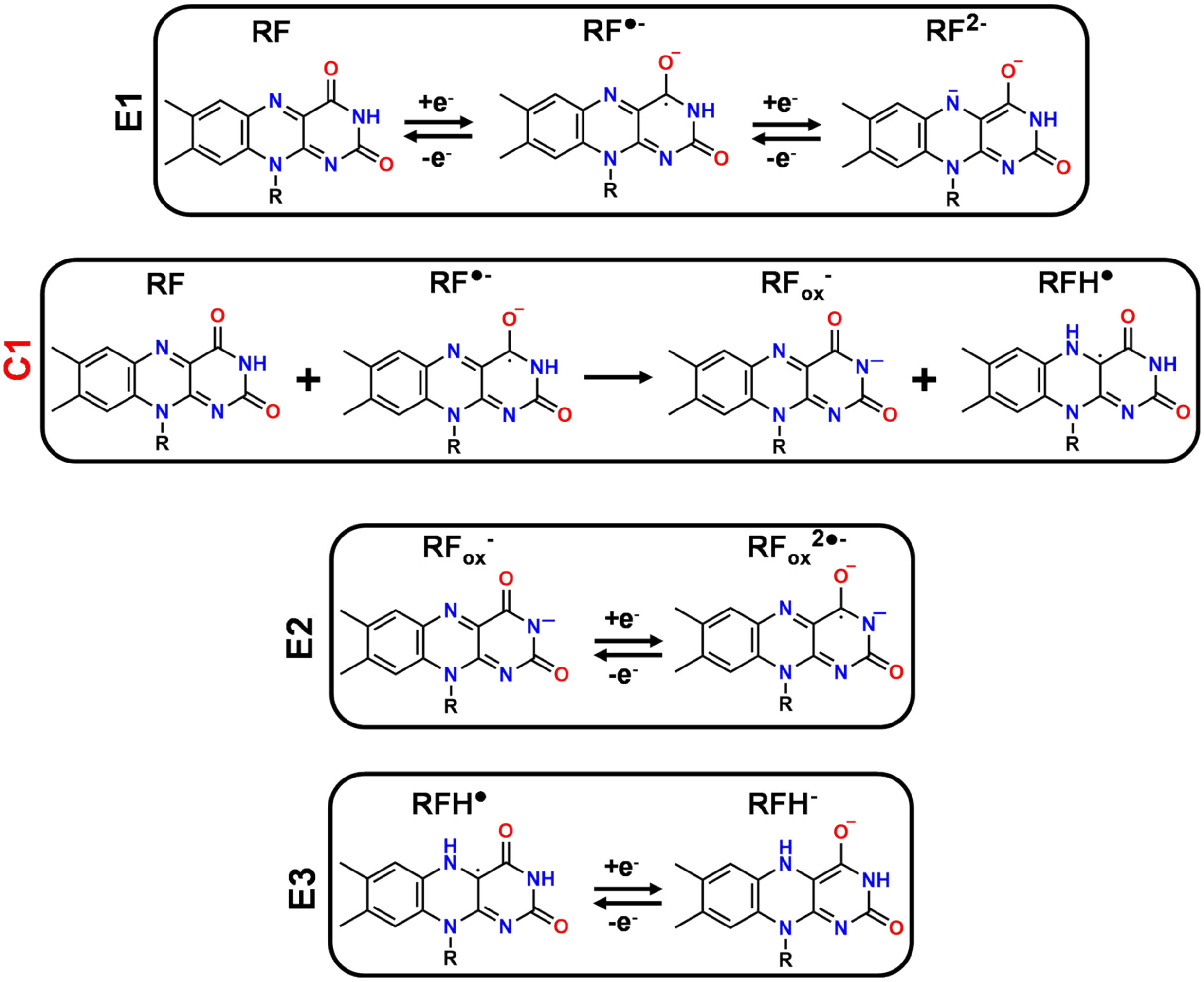

Scheme S5: Depictions of various side reactions that occur when reducing RF in non-aqueous solvents.

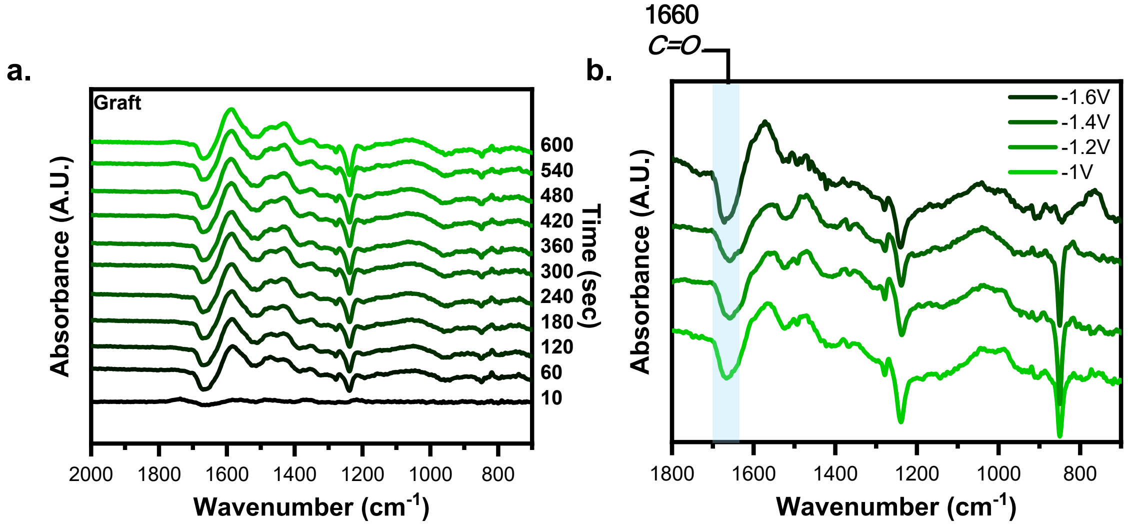

Figure S9: **(a)** Time-resolved spectra taken during the electrografting of MFD-Dz. **(b)** Final spectra taken of the electrografting of MFD-dz onto a graphene-on-gold electrode performed at varying potentials in 0.1 M $TBAPF_6$ in MeCN. The background spectra were taken at 0.4 V under argon.

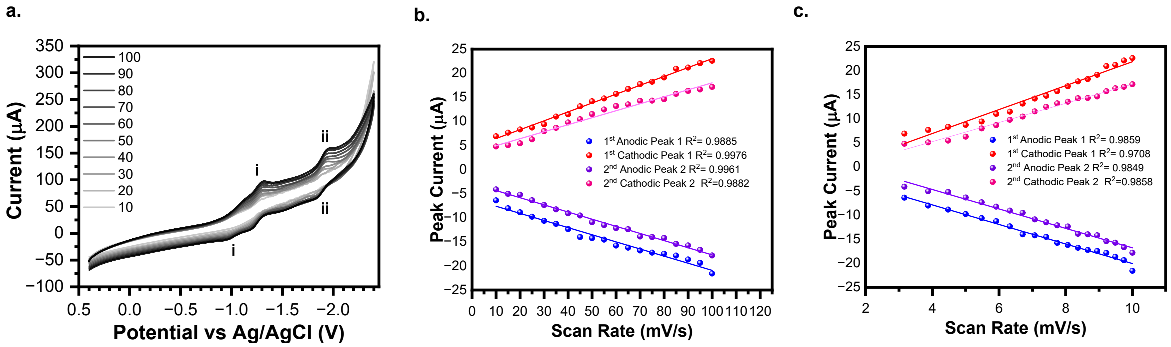

Figure S10: **(a)** CVs of the MFD-modified graphene-gold at different scan rates in argon purged blank 0.1 M $TBAPF_6$ MeCN solution. A plot of the peak current at both redox peaks for AQ vs. **(b)** scan rate and **(c)** the square-root of the scan rate.

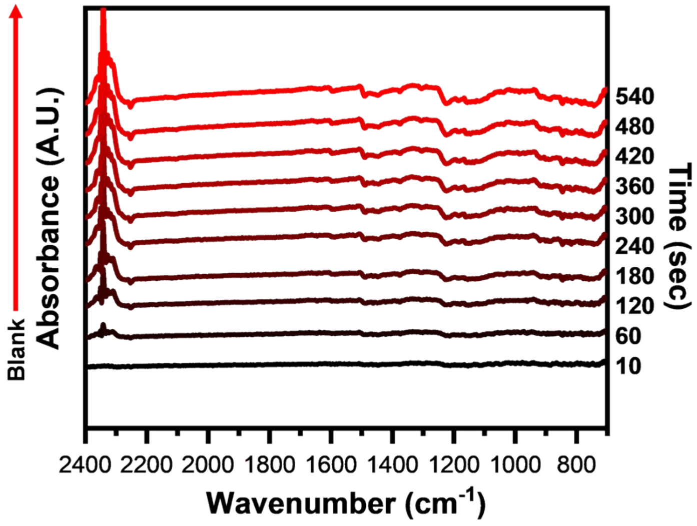

Figure S11: Time-resolved spectra of attempting a CO2 capture by holding -1.5 V for 10 min using a MFD-modified electrode.

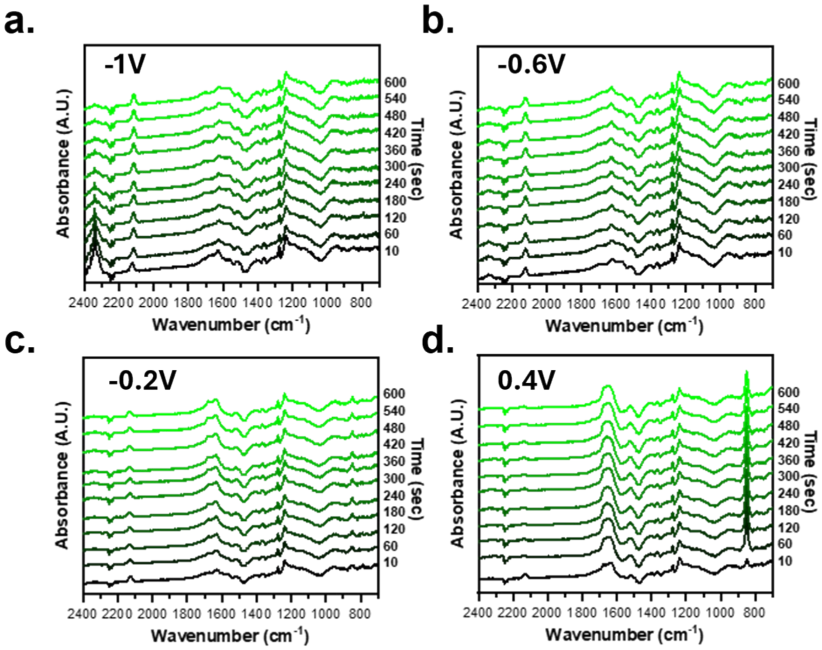

Figure S12: Time-resolve spectra of applying different releasing potentials to CO_2-bound MFD-modified electrodes under argon purge. The releasing potentials used are **(a)** -1 V, **(b)** -0.6 V, **(c)** -0.2 V, **(d)** 0.4 V.

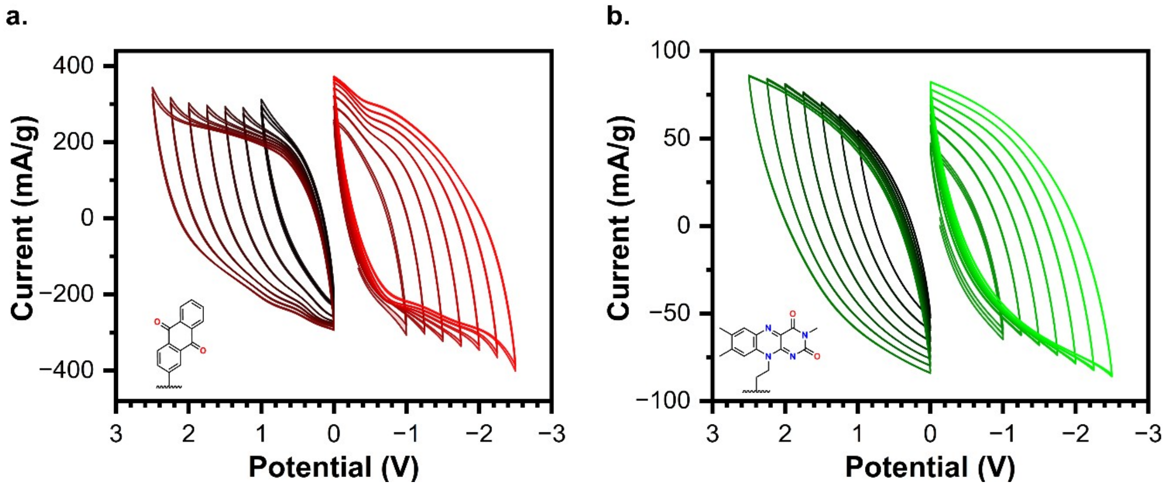

Figure S13: Cyclic voltammetry of 2-electrode flow cell with expanding voltage window from 0.5 V to 2.5 V, both positive and negative for **(a)** AQ-functionalized and **(b)** MFD-functionalized electrodes.

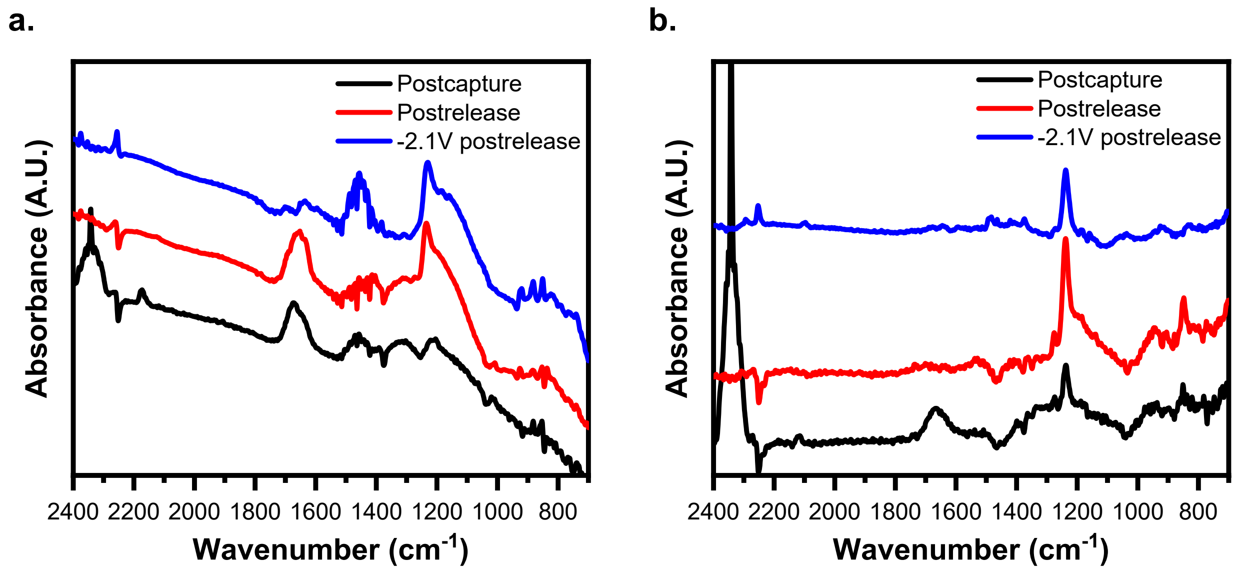

Figure S14: Spectra of **(a)** AQ-modified and **(b)** MFD-modified electrodes after ambient capture, after releasing CO_2 by applying 0.4 V, and applying -2.1 V after release.

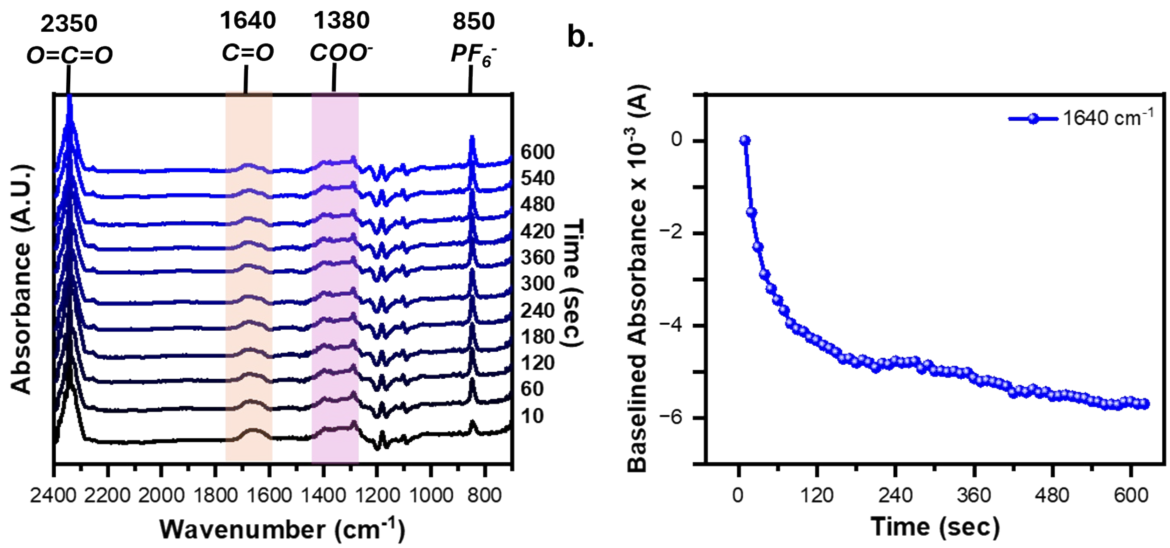

Figure S15: **(a)** Time-resolve spectra were taken at OCP immediately after CO2RR using an unmodified electrode and **(b)** tracking the peak height of the 1640 cm-1 peak for the duration of CO2RR. The background of the spectra was taken at -2.1 V under argon.

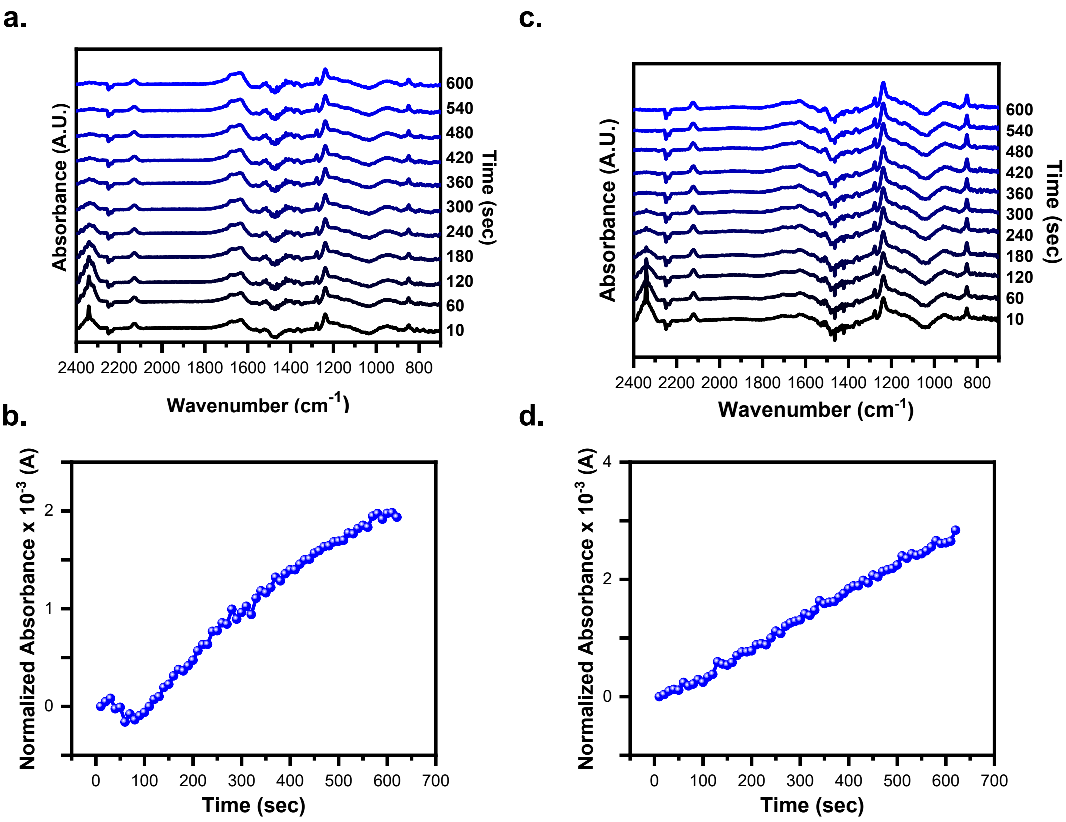

Figure S16: **(a)** Time-resolve spectra were taken at OCP for 10 min after ambient capture using an **(a)** AQ-modified electrode and **(b)** MFD-modified electrode. Tracking the peak height of the 1630 cm^{-1} during the OCP for **(c)** AQ-modified electrode and **(d)** MFD-modified electrode. The background of the spectra was taken at -2.1 V under argon.

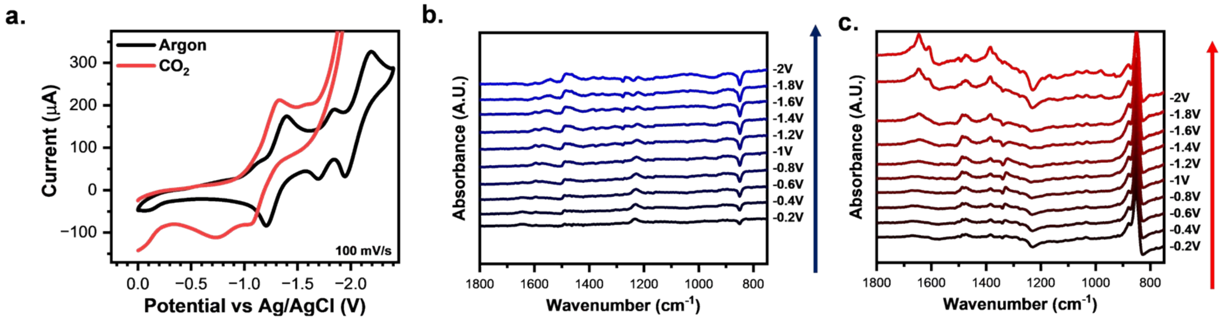

Figure S17: CVs and EC-SEIRAS characterization of freely diffusing 2-aminoanthraquinone (2-AAQ) using a graphene-on-gold electrodes in 0.1M $TBAPF_6$ MeCN. **(a)** CVs of 2-AAQ in a 0.1M $TBAPF_6$ MeCN under argon and CO_2 purge. Potential-dependent spectra 2-AAQ under **(b)** argon or **(c)** CO_2 purge. The background spectra were taken at 0.4 V under argon and CO_2 separately.

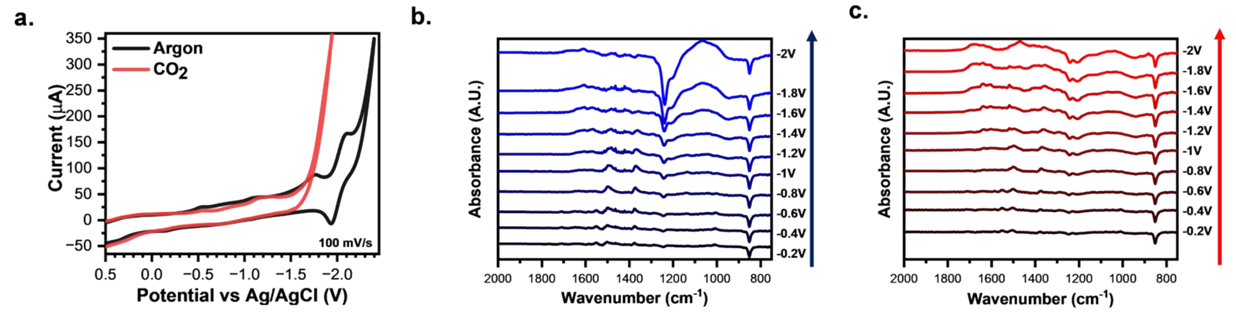

Figure S18: CVs and EC-SEIRAS characterization of freely diffusing AMFD using graphene-on-gold electrodes in 0.1M $TBAPF_6$ MeCN. **(a)** CVs of AMFD in a 0.1M $TBAPF_6$ MeCN under argon and CO_2 purge. Potential-dependent spectra AMFD under **(b)** argon or **(c)** CO_2 purge. The background spectra were taken at 0.4 V under argon and CO_2 separately.

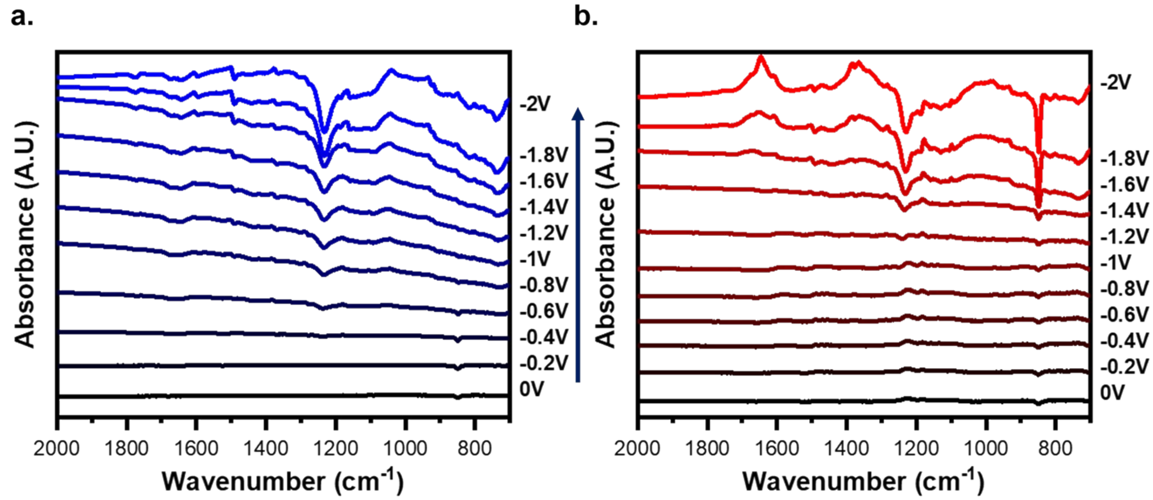

Figure S19: Potential-dependent spectra unmodified graphene-gold under **(b)** argon or **(c)** CO_2 purge. The background spectra were taken at 0.4 V under argon and CO_2 separately.

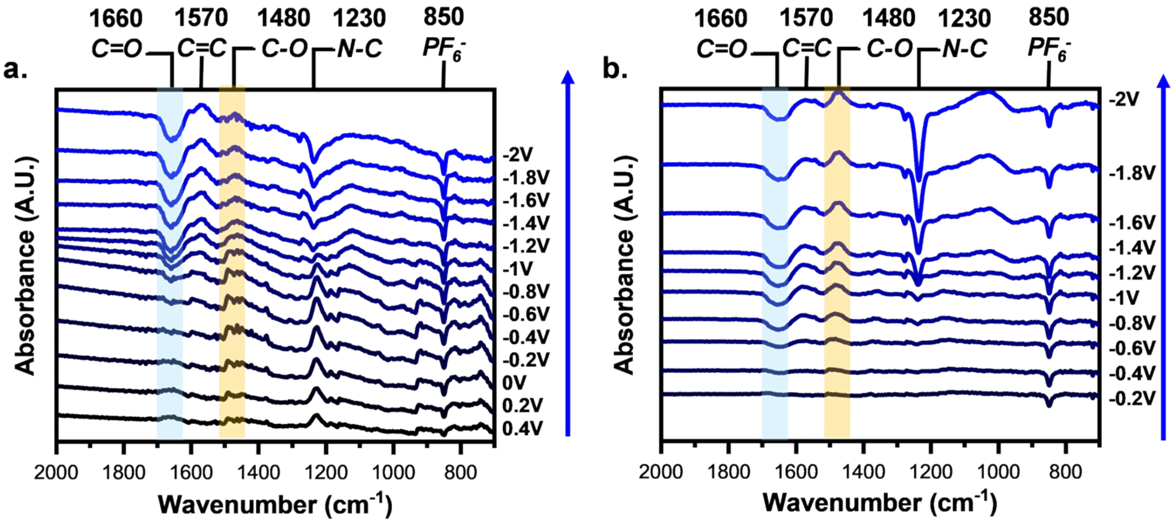

Figure S20: Potential-dependent spectra for **(a)** AQ- and **(b)** MFD-modified graphene-gold under argon in 0.1 M $TBAPF_6$ in MeCN first dried over activated molecular sieves (4 angstrom) followed by fractional distillation from calcium hydride at a high reflux ratio under an argon atmosphere.

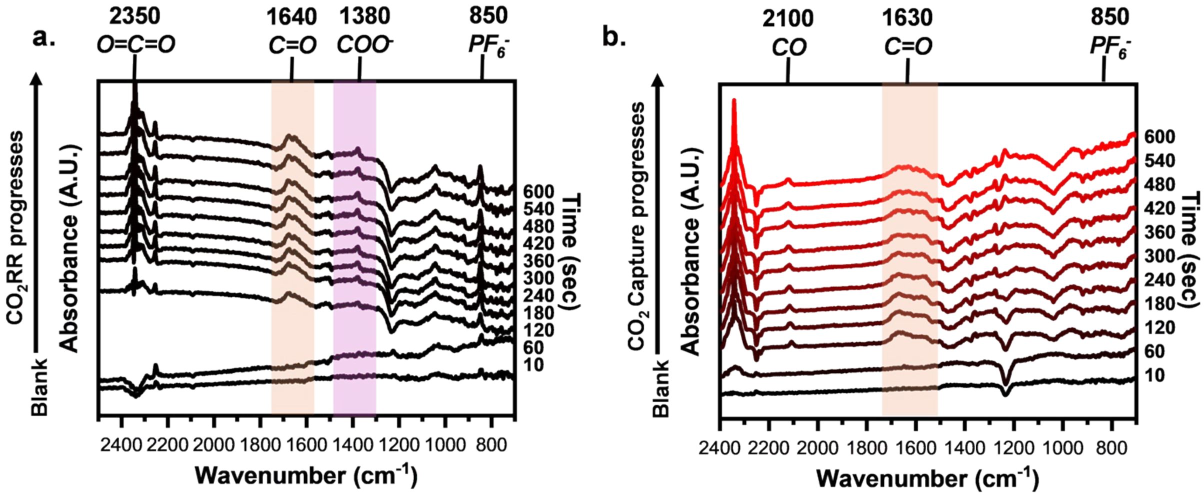

Figure S21: Time-resolve spectra of **(a)** CO_2RR with an unmodified graphene-gold and **(b)** ambient capture of CO_2 with a MFD-modified graphene-on-gold under CO_2 atmosphere in 0.1 M $TBAPF_6$ in MeCN first dried over activated molecular sieves (4 angstrom) followed by fractional distillation from calcium hydride at a high reflux ratio under an argon atmosphere.

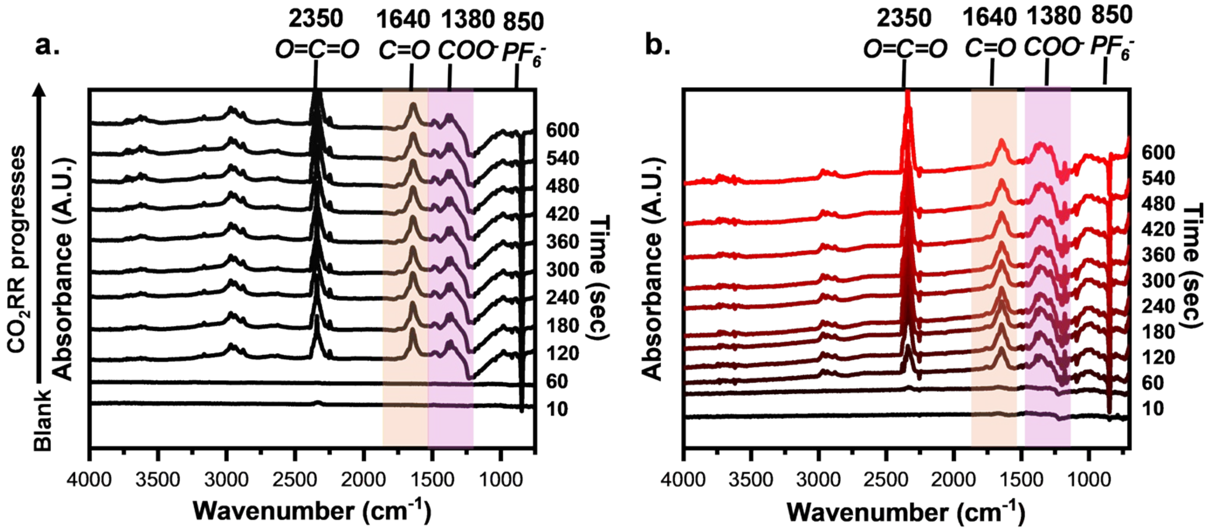

Figure S22: Expanded spectrum from **(a)** Figure 3a and **(b)** Figure S3a to show the region 4000-700 cm^{-1}.

SI.4. CO_2 capture calculations

The amount of CO_2 captured in the flow cell experiments was quantified by integrating the instantaneous %CO_2 flow rate measured from the CO_2 analyzer and converting this to moles of CO_2 using the ideal gas law. We then calculated key performance metrics, including energy

consumption per mole of CO_2 captured (J/mol CO_2) and gravimetric adsorption capacity (mol CO_2/kg of total active electrode material). These calculations are detailed below:

The CO_2 analyzer provides real-time gas-phase CO_2 concentrations in % by volume ($C_{CO_2}(t)$; [%]), which we converted to an instantaneous volumetric flow rate of CO_2 ($Q_{CO_2}(t)$; [cm^3/min]) as a fraction of the total inlet gas flow rate (Q_{total}; 5 cm^3_{STP}/min):

$$Q_{CO_2}(t) = \frac{C_{CO_2}(t)}{100} \times Q_{total} \tag{1}$$

where $C_{CO_2}(t)$ is the instantaneous %CO_2 from the analyzer. We established the baseline CO_2 flow rate ($Q_{baseline}$) by flowing the gas through the flow cell for 30 minutes in the absence of applied bias. This value was measured as 17.87% ± 0.12. We converted flow rate from reported sccm to cm^3/sec before integration. We determined the volume of CO_2 captured (V_{CO_2}; [cm^3]) by the following integration:

$$V_{CO_2} = \int_{t_0}^{t_f} \left[Q_{baseline} - Q_{CO_2}(t)\right] dt \tag{2}$$

We used the ideal gas law to calculate the moles of CO_2 from V_{CO_2}:

$$n_{CO_2} = \frac{V_{CO_2}}{V_{molar}} \tag{3}$$

where V_{molar} is the standard molar volume of CO_2 gas at 1 atm and 298 K (24,450 cm^3/mol CO_2). We used the ideal gas law given the near-ambient operating conditions and the standardization to normal temperature and pressure by the mass flow controller.

We determined the energy used for CO_2 capture ($U_{CO_2,capture}$; [J/mol$_{CO2}$]) from the constant galvanostatic charging:

$$U_{CO_2,capture} = \frac{I \int_{t_0}^{t_f} V(t)dt}{n_{CO_2}} \tag{4}$$

where I is the applied current [A] and V(t) is the voltage [V] response during galvanostatic charging.

We calculated the adsorption capacity (q_{CO_2}; [mol of CO_2/kg of active material]) using the following:

$$q_{CO_2} = \frac{n_{CO_2}}{m_+ + m_-} \tag{5}$$

where m_+ and m_- refer to the mass [kg] of the positive and negative electrode active materials, respectively.

Sample Calculation

1. $$Q_{baseline} = \frac{18}{100} \times 5 \text{ sccm} = 0.9 \text{ sccm} \times \frac{1 \text{ min}}{60 \text{ sec}} = 0.015 \ \text{cm}^3/\text{sec}$$

2. $$V_{CO_2} = \int_{600}^{900} \left[0.015 \ \text{cm}^3/\text{sec} - Q_{CO_2}(t) \right] dt = 2.5 \text{ cm}^3$$

3. $$n_{CO_2} = \frac{2.5 \text{ cm}^3}{24450 \ \text{cm}^3/\text{mol}_{CO_2}} = 1.02 \times 10^{-4} \text{mol}_{CO_2}$$

4. $$U_{CO_2,capture} = \frac{0.005\text{A} \int_{600}^{900} V(t)dt}{1.02 \times 10^{-4} \text{ mol}_{CO_2}} = \frac{13.2 \text{ J}}{1.02 \times 10^{-4} \text{ mol}_{CO_2}} = 1.27 \times 10^5 \ \text{J}/\text{mol}_{CO_2}$$

5. $$q_{CO_2} = \frac{1.02 \times 10^{-4} \text{ mol}_{CO_2}}{6.1 \times 10^{-5} \text{ kg} + 6.3 \times 10^{-5} \text{kg}} = 0.823 \text{ mol}_{CO_2}/\text{kg}$$

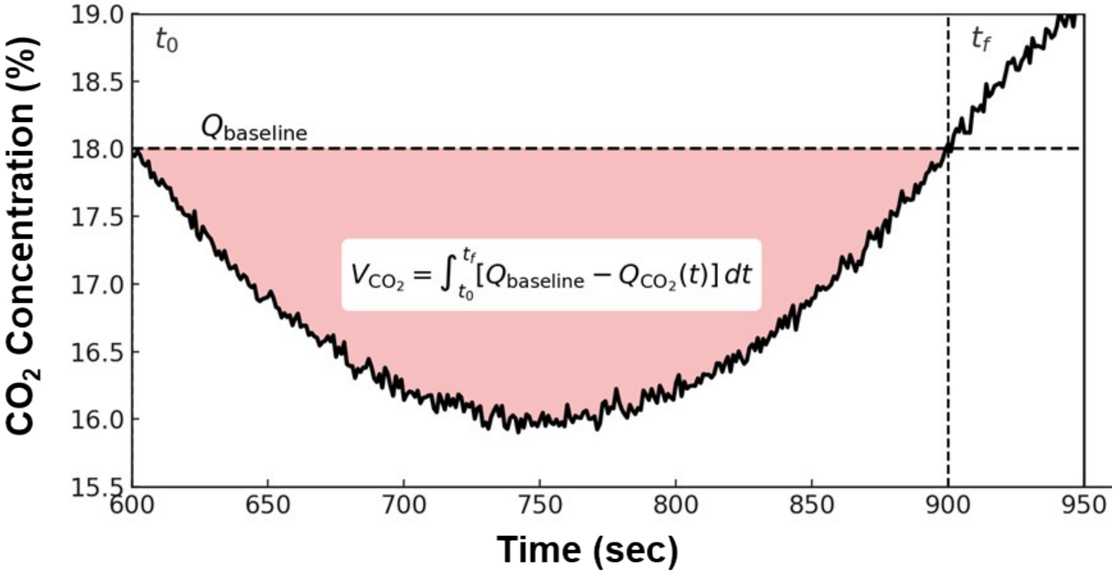

Figure S23: Representative CO_2 concentration profile during the electrochemical flow cell CO_2 capture step from t_0 to t_f, demonstrating the integration method used to calculate the volume of CO_2 removed from the gas stream. The shaded region represents the integrated difference between the baseline and the instantaneous concentration, quantified using Equation 2.
